# Ultra-high resolution X-ray structures of two forms of human recombinant insulin at 100 K

**DOI:** 10.1186/s13065-017-0296-y

**Published:** 2017-08-01

**Authors:** David R. Lisgarten, Rex A. Palmer, Carina M. C. Lobley, Claire E. Naylor, Babur Z. Chowdhry, Zakieh I. Al-Kurdi, Adnan A. Badwan, Brendan J. Howlin, Nicholas C. J. Gibbons, José W. Saldanha, John N. Lisgarten, Ajit K. Basak

**Affiliations:** 1 Biomolecular Research Group, School of Human and Life Sciences, Canterbury Christ Church University, North Holmes Road, Canterbury, Kent, CT1 1QU UK; 2 Department of Crystallography, Biochemical Sciences, Birkbeck College, Malet St, London, WC1E7HX UK; 3 Diamond Light Source Ltd, Diamond House, Harwell Science and Innovation Campus, Didcot, Oxfordshire, OX11 0DE UK; 4 Molecular Dimensions Ltd, Unit 6, Goodwin Business Park, Willie Snaith Road, Newmarket, Suffolk, CB8 7SQ UK; 5 Faculty of Engineering & Science, University of Greenwich (Medway Campus), Chatham Maritime, Kent, ME4 4TB UK; 6 The Jordanian Pharmaceutical Manufacturing Company (PLC), Suwagh Subsidiary for Drug Delivery Systems, P.O. Box 94, Naor, 11710 Jordan; 7 Chemical Sciences Division, Faculty of Health and Medical Sciences, University of Surrey, Guildford, Surrey, GU2 7HX UK; 8 Department of Natural Sciences, School of Science and Technology, University of Middlesex, Hendon Campus, The Burroughs, London, NW4 4BT UK; 9 MRC National Institute for Medical Research, The Ridgeway, Mill Hill, London, NW71AA UK

## Abstract

The crystal structure of a commercially available form of human recombinant (HR) insulin, Insugen (I), used in the treatment of diabetes has been determined to 0.92 Å resolution using low temperature, 100 K, synchrotron X-ray data collected at 16,000 keV (λ = 0.77 Å). Refinement carried out with anisotropic displacement parameters, removal of main-chain stereochemical restraints, inclusion of H atoms in calculated positions, and 220 water molecules, converged to a final value of R = 0.1112 and R_free_ = 0.1466. The structure includes what is thought to be an ordered propanol molecule (POL) only in chain D(4) and a solvated acetate molecule (ACT) coordinated to the Zn atom only in chain B(2). Possible origins and consequences of the propanol and acetate molecules are discussed. Three types of amino acid representation in the electron density are examined in detail: (i) sharp with very clearly resolved features; (ii) well resolved but clearly divided into two conformations which are well behaved in the refinement, both having high quality geometry; (iii) poor density and difficult or impossible to model. An example of type (ii) is observed for the intra-chain disulphide bridge in chain C(3) between Sγ6–Sγ11 which has two clear conformations with relative refined occupancies of 0.8 and 0.2, respectively. In contrast the corresponding S–S bridge in chain A(1) shows one clearly defined conformation. A molecular dynamics study has provided a rational explanation of this difference between chains A and C. More generally, differences in the electron density features between corresponding residues in chains A and C and chains B and D is a common observation in the Insugen (I) structure and these effects are discussed in detail. The crystal structure, also at 0.92 Å and 100 K, of a second commercially available form of human recombinant insulin, Intergen (II), deposited in the Protein Data Bank as 3W7Y which remains otherwise unpublished is compared here with the Insugen (I) structure. In the Intergen (II) structure there is no solvated propanol or acetate molecule. The electron density of Intergen (II), however, does also exhibit the three types of amino acid representations as in Insugen (I). These effects do not necessarily correspond between chains A and C or chains B and D in Intergen (II), or between corresponding residues in Insugen (I). The results of this comparison are reported.

**Electronic supplementary material:**

The online version of this article (doi:10.1186/s13065-017-0296-y) contains supplementary material, which is available to authorized users.

**Graphical abstract:**
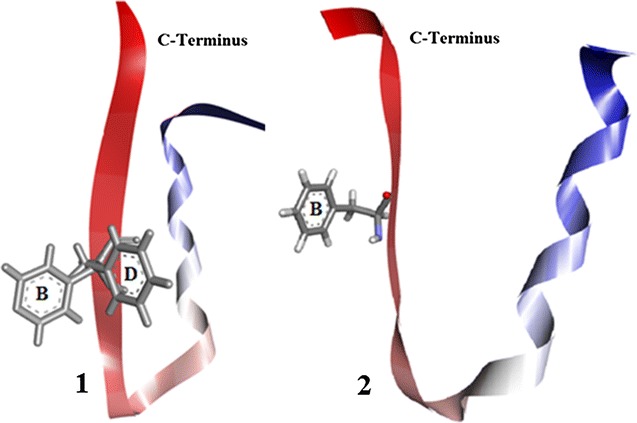
Conformations of PheB25 and PheD25 in three insulin structures: implications for biological activity? Insulin residues PheB25 and PheD25 are considered to be important for insulin receptor binding and changes in biological activity occur when these residues are modified. In porcine insulin and Intergen (II) PheB25 adopts conformation B and PheD25 conformation D. However, unexpectedly PheB25 in Insugen (I) human recombinant insulin adopts two distinct conformations corresponding to B and D, Figure 1 and PheD25 adopts a single conformation corresponding to B not D, Figure 2. Conformations of this residue in the ultra-high resolution structure of Insugen (I) are therefore unique within this set. Figures were produced with Biovia, Discovery Studio 2016.

## Introduction

**Fig. 1:**
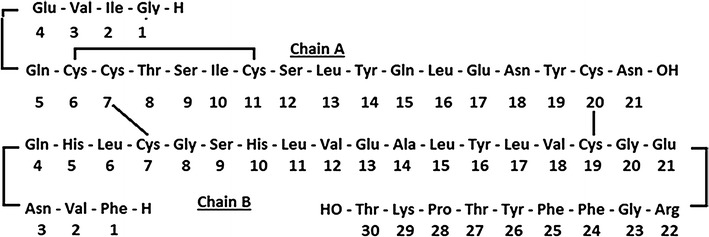
Insugen (I) HR insulin: amino acid sequence. In the porcine insulin sequence ThrB30 is mutated to Ala

A definitive account of the 1.5 Å resolution structure (PDB 4INS) of hexagonal porcine insulin, which differs in sequence by only one amino acid at B30 (and D30) from human insulin ( Fig. 1), was published by Baker et al. [1].

Success in the use of pig insulin to control diabetes ultimately lies in its ability to mimic the activity of the human form, which is a consequence of near perfect structural isomorphism. However, the use of non-human forms of insulin to control diabetes is known to lead to both allergic reactions and other complications resulting from antibody production in some patients [2]. For this reason the use of recombinant forms of human insulin which have now been developed is becoming more commonplace, on the assumption that their structure–function properties are even more closely related to the natural hormone. There are 2 independent molecules in the asymmetric unit of the crystal structure of hexagonal porcine insulin [1]: molecule 1 comprising peptide chains A1 and B1, and molecule 2, comprising peptide chains A2 and B2 (the 4 chains are now usually designated A, B, C and D). Peptide chains A and C are identical in sequence, as are chains B and D. Chains A and B, and chains C and D are linked by disulphide bridges Cys7A–Cys7B, Cys7C–Cys7D, Cys20A–Cys19B and Cys20C–Cys19D, respectively. Chain A also has an internal stabilizing disulphide bridge Cys6A–CysA11 and there is a corresponding S–S bridge in chain C, Cys6C–CysC11. As shown in Additional file 1: Figure S1 there are 3 AB and 3 CD dimers in the unit cell grouped around a crystallographic threefold axis. In the 2Zn crystals, three non-crystallographic insulin dimers are assembled around two Zn ions on the threefold axis. Each Zn ion is coordinated to three symmetry-related Nε atoms of HisB10 and to three water molecules. Water oxygen atoms (282) were also assigned and included in the refinement which converged to a value of R = 0.153 for 10,119 significant I_obs_(hkl). Seven of the amino acid side-chains were assigned less ordered conformations, refined with separate atomic coordinate sets and occupancy factors. Commercial human recombinant insulin is now available from several sources. The present study describes the ultra-high resolution (0.92 Å) low temperature structure of Insugen (I) human recombinant insulin, Fig. 1 and Additional file 1: Figure S2a. The unpublished structure of a second recombinant form of human recombinant insulin, from Intergen, at the same resolution, deposited as structure 3W7Y in the Protein Data Base (in June 2013) shows a number of surprising differences when compared with the Insugen (I) structure reported here. These two structures will be referred to as Insugen (I) and Intergen (II). The Insugen (I) and Intergen (II) 2Zn hexagonal HR insulin structures are predominantly isomorphous with that of porcine 2Zn insulin [1]. In both of these new structures the A and B-chains of molecule 1 are in the T-state [3].

## Implications for biological activity

**Fig. 2:**
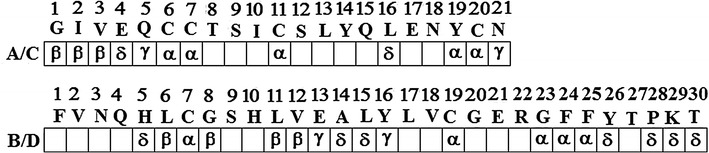
Analysis of residues in the porcine insulin structure of Baker et al. [1] which may be important factors involved in the biological activity. α indicates most likely and γ is least likely to be active. The positionally invariable cysteines that form the disulphide bridges are also included as being very likely to be involved, rated α

HR insulin, Fig. 1, is currently used by the majority of insulin dependent diabetic patients, porcine insulin having been phased out some years ago [2]. The safe therapeutic use of genetically engineered human insulin depends on its structure being absolutely identical to that of the natural molecule, thereby reducing the possibility of complications resulting from antibody production. It has been noted that the use of human recombinant insulin in combination with other drugs may blunt the signs and symptoms of hypoglycaemia [2]. It has been reported [4] that several regions of the insulin molecule are closely related to its biological activity. These include: (a) the positions of the Cys residues that form disulphide bridges; (b) the N-terminal (A1–A5) of the A-chain; moreover the hydrophobic core of vertebrate insulins contains an invariant isoleucine residue at position A2. Lack of variation may reflect this side-chain’s dual contribution to structure and function: IleA2 is proposed both to stabilize the A1–A9 α-helix, see Fig. 4b, and to contribute to a “hidden” functional surface exposed on receptor binding. In fact GlyA1 and IleA2 are stabilized by a network of aqueous H-bonds involving some 18 water molecules in Insugen (I) (see “Results”; Additional file 1: Figure S5a). Also in “Results”, Additional file 1: Figures S5b, c show similar networks in Intergen (II) using the deposited 3W4Y and porcine insulin using the deposited 4INS pdb file. Additional file 1: Figures S5c, d and e show end on views of these networks. Substitution of IleA2 by alanine results in segmental unfolding of the A1–A8 α-helix, lower thermodynamic stability and impaired F binding [5]; (c) C-terminal (A16 and A19–21) regions of the A-chain; (d) regions B5–B8, B11–B16 and B23–26 in the B-chain; (e) moreover crystallographic analysis of the insulin molecule has suggested that the structure comprising both ends of the A-chain (GlyA1, GlnA5, ThrA19 and AsnA21) plus B-chain residues ValB12, ThrB16, GlyB23, PheB24 and PheB25 is important for insulin receptor binding [6]; (e) in addition to the invariant cysteines, only ten amino acids (GlyA1, IleA2, ValA3, TyrA19, LeuB6, GlyB8, LeuB11, ValB12, GlyB23 and PheB24) have been fully conserved during vertebrate evolution [7]; this observation supports the hypothesis derived from alanine-scanning mutagenesis studies that five of these invariant residues (IleA2, ValA3, TyrA19, GlyB23, and PheB24) interact directly with the receptor and five additional conserved residues (LeuB6, GlyB8, LeuB11, GluB13 and PheB25) are important in maintaining the receptor-binding conformation [7]. Baker et al. [1] in the definitive account of the 1.5 Å X-ray structure of 2Zn porcine insulin, concluded that the major flexibility observed at the A-chain N terminus residues A1–A6, and the B-chain C terminus residues B25, B28, B29 and B30 may be important for the expression of insulin activity, especially in view of the rigidity of the rest of the structure. Baker et al. [1] also point out that B25.1 Phe (PheB25) is turned in towards the A-chain whereas B25.2 Phe (PheD25) turns out away from the A-chain. A summary of the residues involved in these considerations of biological activity is given below in Fig. 2. Each residue of interest has been ranked according to the number of times it appears in the discussion: α (mentioned 4 times) to δ (mentioned once). Residues left blank in Fig. 2 are not thought to affect the biological activity. Positionally invariable cysteines forming the disulphide bridges have been designated α.^1^

See also “General comments”, “Peptide side chain electron density and conformations in Intergen (II) [PDB 3W7Y]”, “Comments on the solvated propanol in Insugen (I)”, “PheB24 and PheB25 in Insugen (I) and Intergen (II)” for further discussions of the implications of structure for biological activity.

## Materials and methods

### Materials Insugen (I)

Human recombinant insulin (Insugen-30/70) was supplied by Biocon (India) Ltd. See Additional file 1: Table S1a. Human recombinant insulin, Intergen (II) was produced by the INTERGEN Company and purchased by Sakabe [8] from the SEIKAGAKU Company. Details are to be found in Additional file 1: Figure S2b. Other chemicals including HCl, zinc acetate, acetone, trisodium citrate and NaOH were purchased from Fisher Scientific (UK) and Sigma-Aldrich (UK).

### Crystallization

#### Crystallization of Insugen (I)

The crystals were prepared at room temperature by a batch method similar to that of Baker et al. [1], modified as follows: 0.01 g of insulin as a fine powder was placed in a clean test tube; 1 mL of 0.02 M HCl was added to dissolve the protein; on addition of 0.15 mL of 0.15 M zinc acetate the solution became cloudy due to precipitation of the protein; 0.3 mL of acetone and then 0.5 mL of 0.2M trisodium citrate together with 0.8 mL of water were added and the solution became clear; the pH was checked and increased with NaOH to a pH between 8 and 9 for different batches, thus ensuring complete dissolution. It was then adjusted to the required value of pH 6.3. If any slight turbidity occurred, it was removed by warming the solution. The solution was then filtered using a Millipore membrane/acetate cellulose acetate filter. This removes any nuclei which will encourage precipitation or formation of masses of small crystals.

The solution was then warmed to 50 °C by surrounding the test tube with preheated water in a Dewar. This allowed the solution to cool slowly to room temperature. The test tube was lightly sealed with cling film; crystals formed within a few days and were of a suitable size for X-ray diffraction within 2 weeks; the test tube containing crystals was kept at 4 °C prior to data collection. The crystal used for data collection was about 0.2 mm^3^.

#### Crystallization of Intergen (II)

The following details were supplied by Sakabe [8]. In contrast to the Insugen (I) crystals, Intergen (II) crystals were grown using the vapour diffusion hanging drop method at 293 K. The reservoir solution contained 0.1 M sodium citrate, and 22% (w/v) DMF, and 0.08% (w/v) zinc chloride, pH 8.67 while the protein solution was insulin, Intergen (II) dissolved in 0.02 N HCl to a final concentration of 10 mg/mL. The starting volume of the reservoir solution was 1 mL, and the volume of the drop was 20 μL of protein and reservoir solution in a 1:1 ratio. In 4 or 5 days, crystals were observed to have formed, and after 10 days to 2 weeks, insulin crystals of a size suitable for X-ray diffraction studies were present, typically about 0.5 mm × 0.5 mm × 0.3 mm. The crystal used for 3W7Y data collection was about 1.2 mm × 0.7 mm × 0.5 mm [8].

### X-ray data collection

#### Insugen (I) crystal at Diamond Light Source, MX beamline I02

Crystals grown at room temperature were passed through a 30% glycerol solution, prepared in mother liquor, prior to cryo cooling in liquid nitrogen. Crystals were screened with three test shots, separated by 45° using 0.5 s exposure and 0.5° oscillation. Data were collected at 16,000 keV (λ = 0.77 Å) and 100 K with the Pilatus 6 M detector as close to the sample as possible (179.5 mm). The EDNA strategy [9] was used to obtain a start angle and 180° of data were collected with 0.1° oscillation and 0.1 s exposure. The resolution of useful diffraction data achieved and used for structure analysis was 0.92 Å. The spacegroup is H3 (146) and the unit cell is a = b = 81.827 Å, c = 33.849 Å, α = β = 90° γ = 120°. Further details can be found in Additional file 1: Table S1.

#### X-ray data collection for Intergen (II) crystal at the Photon Factory beamline BL-6C (Ibaraki, Japan)

The following details were supplied by Sakabe [8]. A synchrotron data set to 0.7 Å was collected at the Photon Factory beamline BL-6C using wavelength λ = 0.97974 Å. Data were measured on a specially designed Weissenberg type instrument known as “Galaxy”, employing a fully automated high speed imaging plate detector. The detector comprised a vertically focussing 1 m long bent mirror of Pt-coated fused silica at a distance of 21 m from the SR source point and 7 m from the focal point. The low resolution limit was 50.0 and high resolution limit 0.7 Å; the number of reflections observed was 91.73%; R_merge_ for I_obs_ = 0.05579 for 57006 hkl’s. The resolution of useful diffraction data achieved and used for structure analysis was 0.92 Å [10–14]. The space group is: H3 (146); the unit cell is: a = b = 81.120 Å, c = 33.930 Å, α = β = 90° γ = 120°.

### X-ray data processing for Insugen (I) crystal

Manual processing of the data was carried out using XDS [15] to integrate and Aimless [16] to scale and merge intensities. The purpose of manual scaling was to optimise the included data to maximise the final resolution to 0.92 Å.

### Structure solution and initial refinement

#### Insugen (I)

Molecular replacement was carried out with the published structure 3E7Y as a search model in the program MOLREP [17], followed by ten cycles of least squares refinement using the program REFMAC [18].

Further details can be found in Additional file 1: Table S1.

### Presence of Zn in the Insugen (I) Crystal

**Fig. 3:**
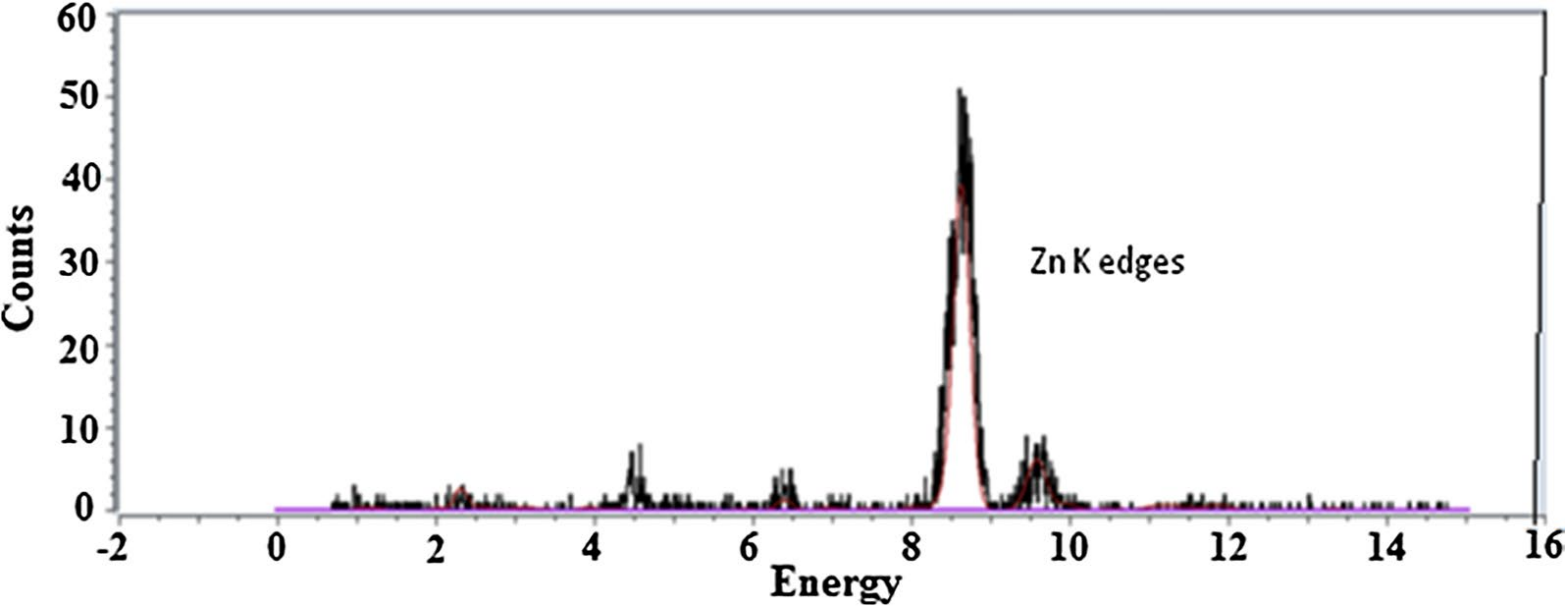
Fluorescence spectra collected from a crystal of HR insulin, Insugen (I), to confirm the presence of zinc

A fluorescence mca scan, Fig. 3, was carried out to confirm the presence of zinc in the crystals.

### Model building and further least squares refinement

#### Insugen (I)

Model inspection and rebuilding were performed using the program WinCoot 0.7 [19] and further isotropic refinement was carried out with the program PHENIX [20]. Water molecules were added at the end of refinement using the automated method provided in PHENIX. Refinement of the Insugen (I) crystal structure was continued using the program SHELX-97 interfaced with SHELXPRO [21]. This facilitated the overall inclusion of H atoms and use of anisotropic temperature factors for the non-H atoms. For the protein structure H atoms initially assigned in calculated positions were refined with isotropic thermal parameters. H atoms were not assigned to the waters. During the course of this phase of the analysis several residues were observed in the electron density to have ordered or clear double conformations which were built into the structure and their relative occupancies were included in the refinement summing to 1.0. At the end of the SHELXPRO refinement the R factor and R_free_ (all data) were 0.108 and 0.146, respectively. The program MolProbity [22] was used for structure validation. Inspection of the Ramachandran plot revealed that 97.53% of the residues are in allowed regions. All coordinates and data have been deposited in the Protein Data Bank, with identification code 5E7W. The final statistics of refinement are summarized in Table 1.

**Table 1:** Data-collection and final refinement statistics

	Insugen (I) (Biocon)	Intergen (II) (Intergen)
Space group	H3	H3
Unit-cell parameters	a = b = 81.8270 Å	a = b = 81.120 Å
	c = 33.8490 Å	c = 33.930 Å
	α = β = 90°	α = β = 90°
	γ = 120°	γ = 120°
V_M_ (Å^3^ Da^−1^)	1.97	1.94
Solvent content (%)	22 (223 waters)	25 (275 waters)
Resolution range (Å)	0.92–13.64	0.92–20.0
No. of measurements	294,902	
No. of unique reflections	50,178	54,114
Completeness (%)	100	98.59
Multiplicity	5.0 (4.1)	4.2
R_merge_^a^	0.028	0.05579
I/σ(I)_mean_	20.2 (4.0)	
R factor	0.1112	0.162
R_free_	0.1446	0.180
Residues in allowed regions of Ramachandran plot (%)	97.53	96.81
R.m.s.d. bonds (Å)	0.017	0.006
R.m.s.d. angles (°)	2.65	1.187
All-atom B_average_ (Å^2^)	17.947	14.737

Values in parentheses are for the highest resolution shell

^a^ ${\text{R}}_{\text{merge}} = \sum_{\text{hkl}} \sum_{\text{i}} \left| {{\text{I}}_{\text{i}} ({\text{hkl}}) - \langle {\text{I}}({\text{hkl}})\rangle } \right|/\sum_{\text{hkl}} \sum_{\text{i}} \left| {{\text{I}}_{\text{i}} ({\text{hkl}})} \right|$ where I_i_(hkl) and 〈I(hkl)〉 are the observed intensity and mean intensity of related reflections respectively

#### Model building and further least squares refinement for Intergen (II)

The structure for 3W7Y was determined by molecular replacement and refined using the program REFMAC [18]. Non-hydrogen atoms were refined anisotropically. Several residues were modelled as two clear conformers with complementary occupational parameters having a sum of 1.0. At the end of the refinement the R factor and R_free_ were 0.162 and 0.180, respectively. Inspection of the Ramachandran plot revealed that 96.81% of the residues are in the allowed regions. All coordinates and data are deposited in the Protein Data Bank, with identification code 3W7Y.

## Results

### General comments

Superficially the ultra-high resolution structure of HR insulin (Insugen I), as expected, strongly resembles that of 2Zn porcine insulin (see “Introduction”) having an asymmetric unit with 2 independent molecules: molecule 1, comprising peptide chains A and B; and molecule 2, comprising peptide chains C and D. Peptide chains A and C are identical in sequence, as are chains B and D.

As described below there are significant and interesting differences between the detailed ultra-high resolution structures of Insugen (I) and Intergen (II) and also between the two human recombinant insulin structures and the less detailed porcine insulin [1]. For example in the porcine insulin structure [1] 289 waters were assigned and in Intergen (II) 275. However after intense scrutiny and assessment 220 water molecules have been included and refined in the Insugen (I) structure. Further features of interest in the Insugen (I) structure are: (i) an acetate molecule ACT2101 (or simply ACT) has been assigned in the neighbourhood of Zn2100 in molecule 1 and is in fact coordinated with this Zn. This unexpected feature is described below and is presumably a consequence of the zinc acetate used in the crystallization procedure. The acetate molecule has excellent refinement parameters and geometrical features. To the best of our knowledge acetate has not been assigned to any other published insulin structure; further evidence for this assignment can be found in Additional file 1: Text S1 and Figure S3: (ii) a solvated propanol molecule has been assigned as described below in detail. The propanol molecule POL5001 (or simply POL) forms H-bonds with the prominent Oγ1A of ThrD27 located on the A conformation of ThrD27 which has two clearly defined conformations A and B, of which A has 0.645 occupancy and B 0.355 occupancy. POL is also H-bonded to water 6007. Further evidence for the assignment of propanol can be found in Additional file 1: Text S2 and Figure S4. There is no evidence of propanol solvate close to ThrB27 in chain B which has a single fully occupied conformation (see below). Intergen (II) shows no evidence of either acetate or propanol in the electron density for the deposited 3W7Y structure. To the best of our knowledge solvated propanol has not been reported as present in any other determined insulin structure. Possible origins of the solvated propanol are examined. As discussed below other differences occur between the two human recombinant insulin crystal structures. Such differences may ultimately be of importance with respect to the hormonal and biological activities of these synthetic therapeutics [2].

### Description of the secondary structure regions in Insugen (I)

The ultra-high resolution refinement of HR insulin, Insugen (1) undertaken in the analysis described above has enabled a study of the secondary structure motifs in the insulin molecule to be carried out in detail which exceeds all previous studies.

#### Chain A (Fig. 4a)

**Fig. 4:**
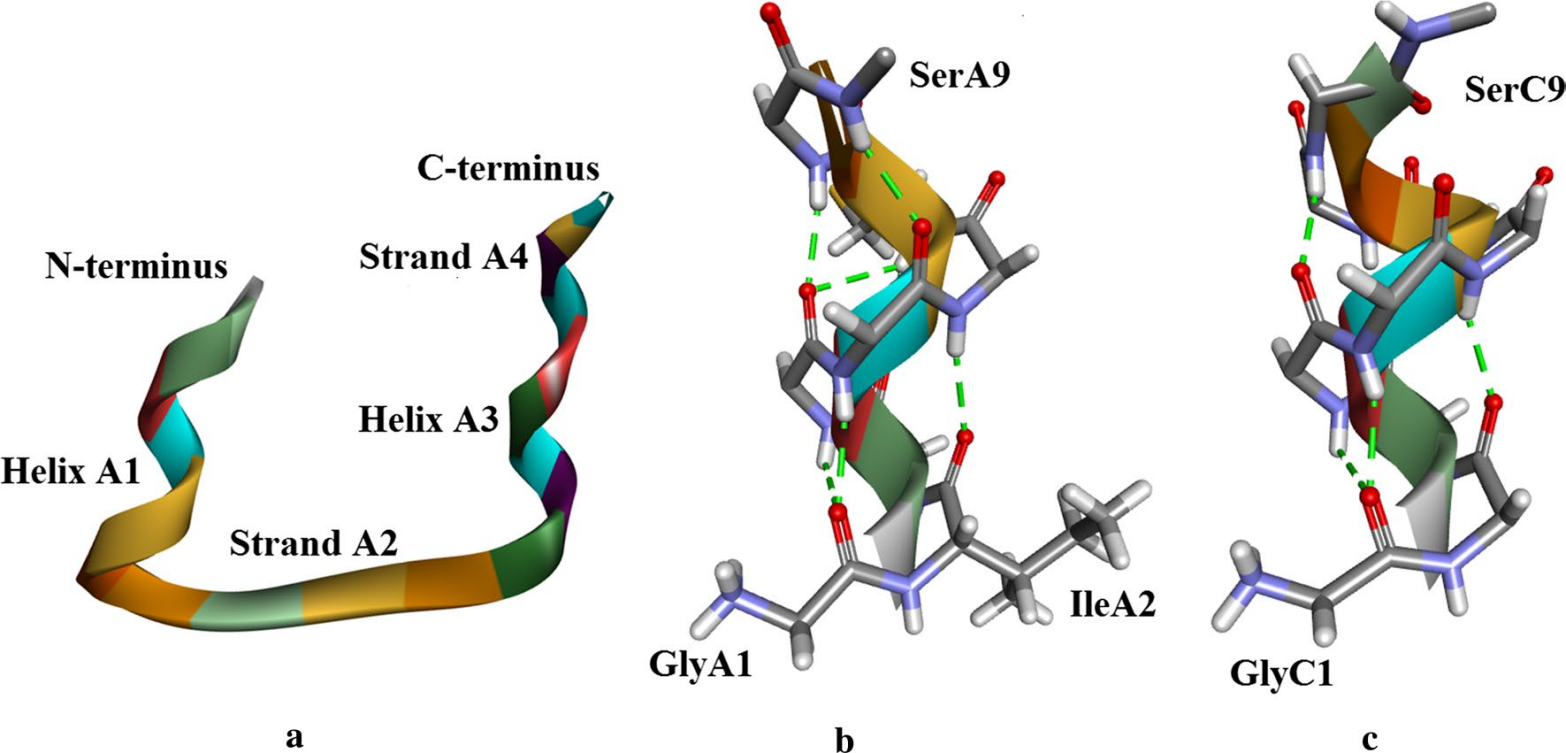
**a** Insugen (I) chain A, secondary structure motifs. **b** Insugen (I) Helix A1: GlyA1–SerA9. With the exception of side-chains GlyA1 and IleA2 which are shown completely, only main chain atoms are shown. H-bonds are shown as *green dotted lines*. Compare with Figure S9a which shows the same region in porcine insulin [1]. **c** Insugen (I) Helix A3: LeuA13–TyrA19. H-bonds are shown as *green dotted lines*. Main chain atoms only are shown. Drawn with Biovia, Discovery Studio 2016 [35]

**Helix A1** (Fig. 4a, b)

**Helix A1**: This involves the first 9 residues GlyA1–SerA9 and comprises about 2 turns of a distorted α-helix. Although GlyA1 involves a bifurcated H-bond and its (φ, ψ) values are indeterminate because it is N-terminal, this residue does seem to be part of the helix. SerA9 is at the C-terminal end of the helix, its side chain H-bonding to the peptide N of IleA10. Details are in Fig. 4b.

**Strand A2** (Fig. 4a)

Strand A2 runs from IleA10–SerA12 forms an anti-parallel sheet with strand B1 in the B-chain (see below). Note there is only one β-bridge, at CysA11.

**Helix A3** (Fig. 4a, c): this secondary structure involves LeuA13–TyrA19 and is a 7 residue 3_10_ helix. The SerA12 side-chain caps the N-terminal end of the helix by H-bonding to the peptide N of GlnA15, whose side-chain in turn forms an H-bond to the N of SerA12. The carbonyl of SerA12 forms the first H-bond of the helix, but the (φ, ψ) values of SerA12 suggest it is part of the preceding strand and not this helix.

**Strand A4** (Fig. 4a): CysA20 and AsnA21 appear to form a mini strand and participate in an anti-parallel sheet with strand B4 (Fig. 6a) in the B-chain strand. The carbonyl oxygen of TyrA19 forms the first H-bond of the strand although it is part of the preceding helix.

#### **Chain C** (Fig. 5a)

**Fig. 5:**
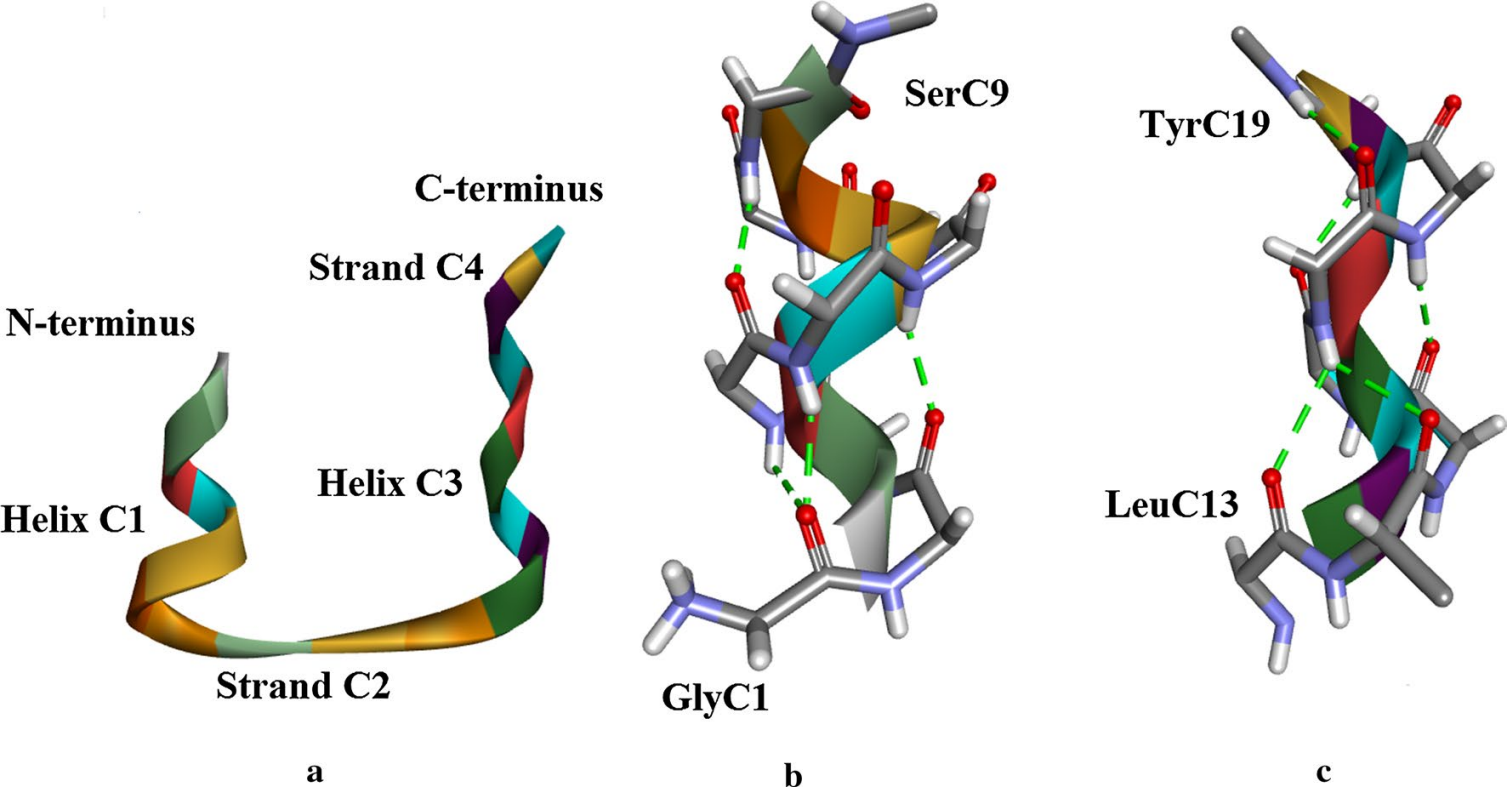
**a** Insugen (I) chain C: secondary structure motifs (compare with Fig. 4a for chain A). **b** Insugen (I) Helix C1: GlyC1–SerC9. H-bonds are shown as *green dashed lines*. Main chain atoms only are shown. Compare with Figure S9b which shows the same region in porcine insulin [1]. **c** Insugen (I) chain C Helix C3: LeuC13–TyrC19. H-bonds are shown as *green dashed lines*. Main chain atoms only are shown. Drawn with Biovia, Discovery Studio 2016 [35]

**Helix C1** (Fig. 5a, b): GlyC1–SerC9 form a 9 residue 2 turn helical structure. The first turn (GlyC1-GluC4) is α-helix, but then GluC4 forms a H-bond with SerC9 (i.e. i to i + 5) creating a much looser turn. Strictly, this is one turn of π-helix. SerC9 terminates the helix by its side chain H-bonding to the peptide N of IleC10.

**Strand C2** (Fig. 5a): IleC10–SerC12 form a strand, based on the (φ, ψ) values. SerC9 is probably not part of this strand as its φ, ψ value is at the edge of the β-strand region (φ = −90^o^,ψ = −164^o^). The strand extends to SerC12.

**Helix C3** (Fig. 5a, c): LeuC13–TyrC19 is a 7 residue 3_10_ helix comprising about 2 turns. SerC12 caps the N-terminus end with its side-chain forming an H-bond with the peptide N of GlnC15, while the side-chain of GlnC15 forms an H-bond with the peptide N of SerC12.

**Strand C4** (Fig. 5a): CysC20 and AsnC21 comprise a mini strand and this forms an anti-parallel sheet with strand D4 in the D-chain (see below).

#### **Chain B** (Fig. 6a)

**Fig. 6:**
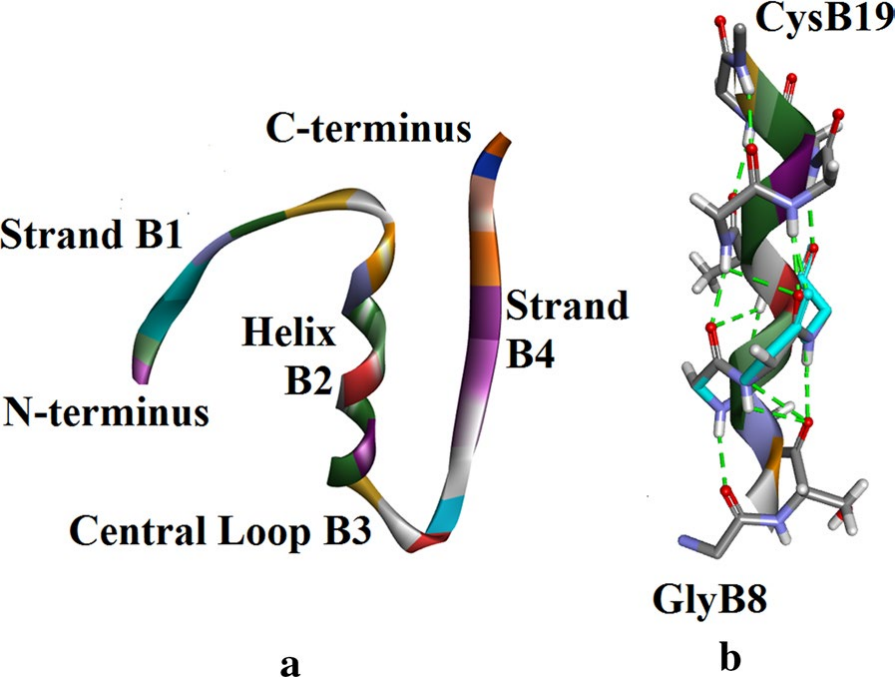
**a** Insugen (I) chain B secondary structure motifs. **b** Chain B Helix B2: GlyB8-CysB19. H-bonds are shown as *green dashed lines*. Main chain atoms only are shown. *Light blue* regions correspond to residues with double side chain conformations. Drawn with Biovia, Discovery Studio 2016 [35]

**Strand B1** (Fig. 6a): This comprises seven residues from PheB1 to CysB7, based on (φ, ψ) values. This strand forms an anti-parallel sheet with the strand A2 in the A-chain.

**Helix B2** (Fig. 6a, b): This extends from GlyB8 to CysB19 (12 residues), about 3-turns of α-helix. Note GlyB8 does not have helical (φ, ψ) values but does have a 3_10_ turn H-bond.

**Central Loop B3** (Fig. 6a): There is a type I turn from GlyB20–GlyB23 and an open α-turn from CysB19 to GlyB23.

**Strand B4** (Fig. 6a): In terms of (φ, ψ) values, this strand could be considered to extend from PheB24 to ThrB30, but in terms of H-bonds in the sheet, it ends at LysB26. It forms an anti-parallel sheet with D4. Note that strands A4 and C4 are part of this four-strand sheet.

#### **Chain D** (Fig. 7a)

**Fig. 7:**
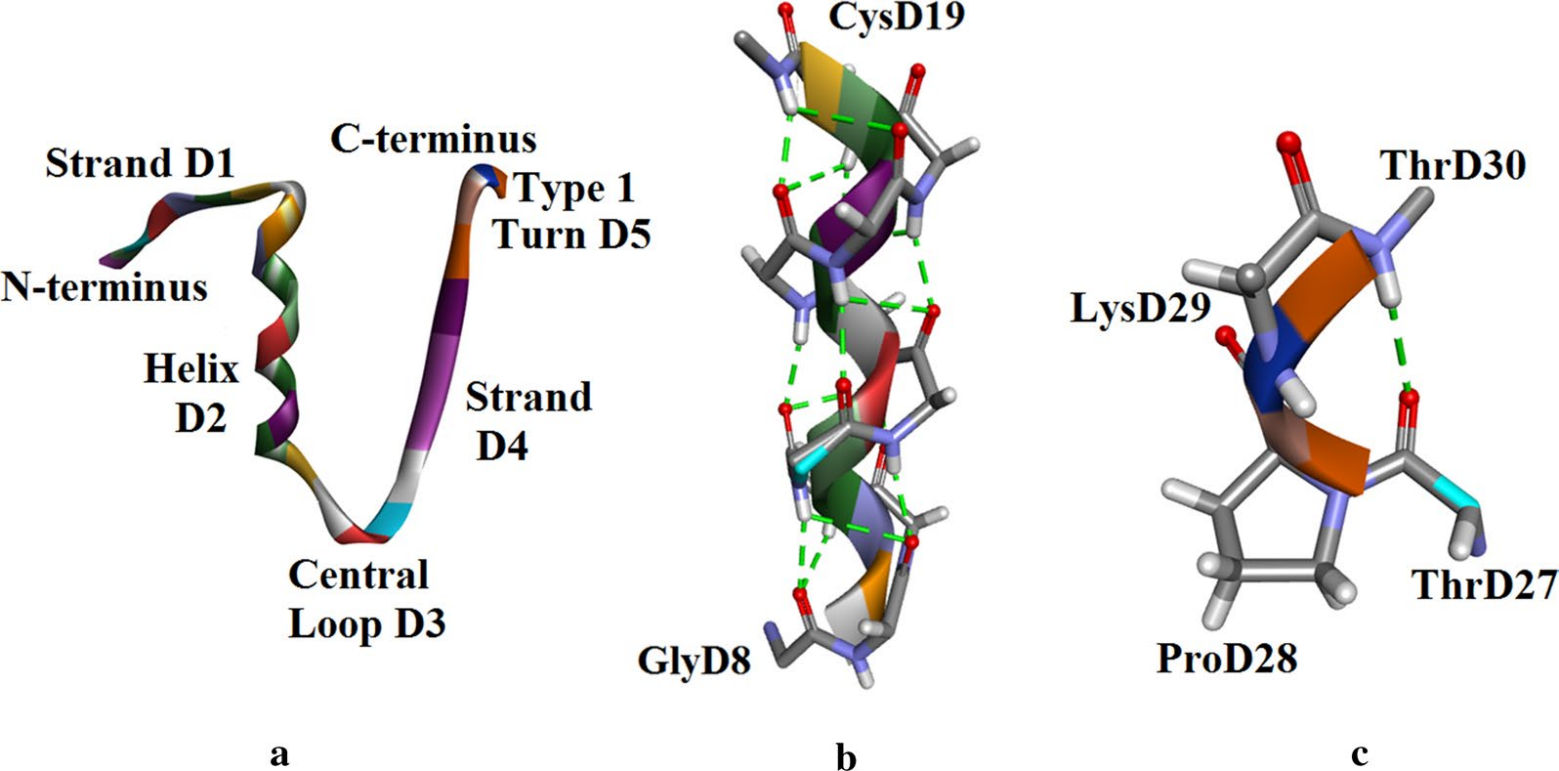
**a** Insugen (I) chain D: secondary structure motifs. **b** Insugen (I) chain D helix D2. H-bonds are shown as *green dashed white lines*. Main chain atoms only are shown. **c** Insugen (I) chain D: type I turn. Main chain atoms only are shown. The H-bond is shown as a *green dashed line*. There is no corresponding secondary structure element in chain B, Fig. 6a. Drawn with Biovia, Discovery Studio 2016 [35]

**Strand D1** (Fig. 7a): Based on (φ, ψ) values this strand comprises seven residues from PheD1 to CysD7. It is perpendicular to strand C2 but does not form a sheet. There is only one H-bond from NH of LeuD6 to CO of CysC6 of chain C which is part of helix C1.

**Helix D2** (Fig. 7a, b): This is a 12 residue α-helix from GlyD8 to CysC19. Note CysD7 is part of strand D1, GlyD8 does not have helical (φ, ψ) values but does have a bifurcated H-bond and CysD19 is helical.

**Central Loop D3** (Fig. 7a): The region CysD19–GlyD23 forms an open-α turn and GlyD20-GlyD23 form a type I turn.

**Strand D4** (Fig. 7a): This extends from PheD24 to TyrD26. It forms a sheet with strand B4 and this sheet also comprises strands A4 and C4.

**Type I Turn D5** (Fig. 7a, c): This is a type I turn and comprises ThrD27, ProD28, LysD29 and ThrD30.

### Solvent molecules

#### Solvated water molecules in Insugen (I)

**Fig. 8:**
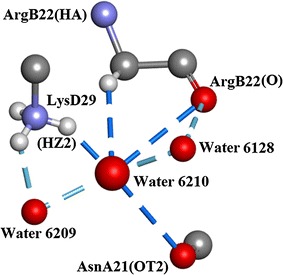
Water 6210 hydrogen bonds to two waters and three amino acid side-chains. Drawn with Biovia, Discovery Studio 2016 [35]

In the crystallographic asymmetric unit a total of 220 water molecule positions were assigned by stereochemical inspection and evaluation of the electron density displayed by WinCoot 0.7 [19]. These were included successfully in the ShelxL refinement with anisotropic thermal displacement parameters. Water H atoms were fixed geometrically. Analysis of the hydrogen bonding properties of the water molecules was carried out using Accelrys Discovery Studio 3 [23] which enabled H-bond geometry to be tabulated. These results are summarised in Table 2 which shows the presence of a variety of H-bond types with acceptable molecular geometry involving different combinations of side-chain–water interactions and water–water interactions. For a given water molecule the number of side-chain–water interactions varies from 0 to 7 and the number of water-water interactions from 0 to 5. A total of 285 side-chain–water H-bonds and 139 unique water–water H-bonds were observed. Figure 8 shows an example of a water molecule, water 6210, having 4 H-bonds to side-chain atoms and 2 H-bonds to other waters (6128 and 6209), denoted by type 4,2 in Table 2.

**Table 2:** Types of H-bond involving water and their numbers: *W–SC* water–side chain, *W–W* water–water

Type of H-bond W–SC, W–W	0,1	0,2	0,3	0,4	0,5	1,0	1,1	1,2	1,3	1,4	2,0	2,1	2,2	2,3
Number N	18	19	6	3	1	24	18	27	6	2	7	14	6	6

Type of H-bond W–SC, W–W	3,0	3,1	3,2	*3,3*	3,4	4,0	4,1	4,2	4,3	5,0	5,1	6,0	7,0
Number N	8	6	6	*2*	2	2	6	2	1	1	2	1	1

Eg 3,3 N = 2 (italicized) means that 2 water molecules have a total of 3 hydrogen bonds to side chain atoms plus 3 hydrogen bonds to another water molecule (6 hydrogen bonds in total)

#### Salt bridges in Insugen (I)

Residues involved in the six salt bridges observed in the Insugen (I) structure are listed in Table 3 together with the corresponding bridge length.

**Table 3:** Insugen (I) residues involved in salt bridge formation

Residue 1	Residue 2	Distance in Å
GLYA1:H0C	GLUA4:OE1	1.94398
ARGB22:HH1	GLUA17:OE2	2.25535
ARGB22:HH2	GLUA17:OE2	2.59606
LYSB29:NZ	THRB30:OT2	2.4885
GLYC1:H0C	GLUC4:OE1	1.79717
ARGD22:HH1	ASNC21:OT1	2.52927

**Fig. 9:**
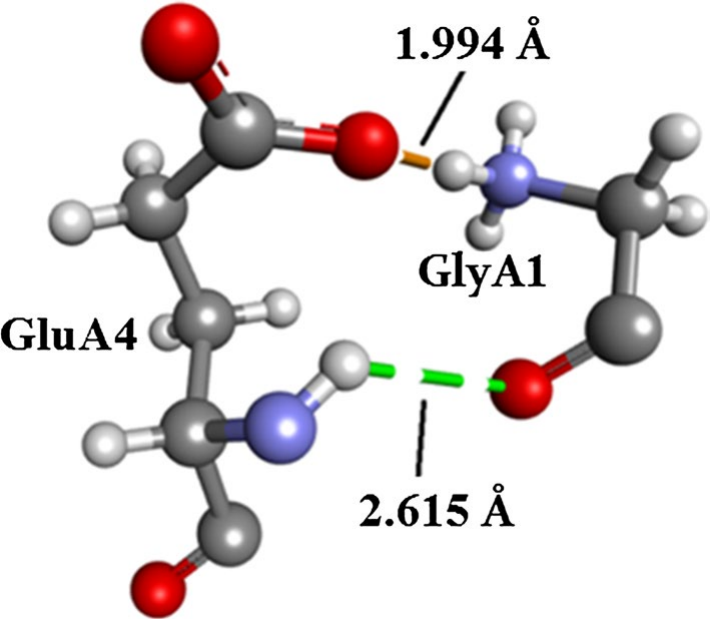
The salt bridge GLYA1:HOC and GLUA4:OE1 in Insugen (I). Relevant distances in Å are indicated. Drawn with Biovia, Discovery Studio 2016 [35]

Figure 9 shows the salt bridge between GLYA1:HOC and GLUA4:OE1.

#### Water–side chain interactions in Insugen (I)

Of the 102 amino acid residues in Insugen (I) a total of 18:2 in both chains A and C; 8 in chain B; and 6 in chain D do not form any hydrogen bond interactions with solvated water molecules. These residues are as follows:

**Table:** 

Chain A	SerA9	**LeuA16**						
Chain B	**LeuB11**	**ValB12**	**AlaB14**	**LeuB15**	**CysB19**	GlyB23	**PheB24**	ThrB30
Chain C	IleC2	**LeuC16**						
Chain D	**LeuD11**	**ValD12**	**AlaD14**	**LeuD15**	**CysD19**	**PheD24**		

The table above indicates the 18 residues in Insugen (I) which do not form any hydrogen bond interactions with solvated water molecules. There are 2 in both chains A and C; 8 in chain B; and 6 in chain D. Entries in bold are common to two chains, either A and C, or B and D.

**Fig. 10:**
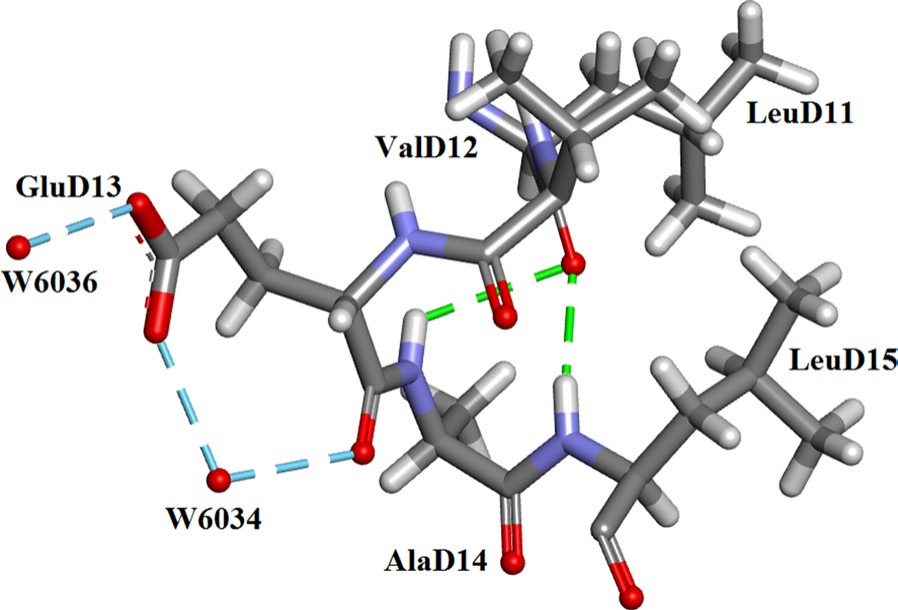
Structure of the Insugen (I) sequence LeuD11–LeuD15. In which only GluD13 forms H-bonds with solvated water molecules. In the Insugen (I) structure only 18 of 102 residues fail to link to solvated water molecules. Drawn with Accelrys Discovery Studio 3 [23] [note Intergen (II) also displays this H-bonding in the LeuD11–LeuD15 sequence]

The residues common to two chains are in bold: Leu16 in both chains A and C are without water interactions as are Leu11, Val12, Ala 14, Leu15 and Cys 19 in both chains B and D. The sequence LeuD11–ValD12–GluD13–AlaD14–LeuD15 is shown in Fig. 10. GluD13 is the only residue in this sequence which forms H-bonds with water molecules i.e W6034 with OE2 and W6036 with OE1.

It is of interest to note that 14 of the 18 residues that do not associate with solvated water molecules are located in α-helical structures. These are: CysA6 (helix A1); LeuA16 (helix A3); LeuB11, ValB12, AlaB14, LeuB15 and CysB19 (helix B2); IleC2 (helix C1); LeuC16 (helix C3); LeuD11, ValD12, AlaD14, LeuD15 and CysD19 (helix D2).

### Survey of the peptide side chain electron density and conformations in Insugen (I) and Intergen (II)

#### Peptide side chain electron density and conformations in Insugen (I)

It is well known that ultra-high resolution protein structures derived from X-ray diffraction data using cryo cooled crystals often reveal amino acid residues which display more than a single ordered conformation. See for example Smith et al. [24] and Addlagatta et al. [25]. When such effects are observed it is possible that the use of these harsh high speed experimental conditions have both caused and allowed these alternative structures to be captured for detailed examination. It is also possible that such alternative conformations may have a bearing on the biological activity of the protein. As described below, the present ultra-high resolution structures of human recombinant insulin Insugen (I) and Intergen (II) both display several amino acid residues having two distinct ordered conformations. As described in detail below the same residues are not necessarily affected in corresponding protein chains in either the Insugen (I) or Intergen (II) structure. Thus, somewhat surprisingly, the disordered regions do not match 1:1 between the two recombinant structures or between corresponding protein chains in the same structure. A detailed analysis and comparison is given below. It is possible that these structural features may affect the biological functions of these recombinant insulins [2].

**Fig. 11:**
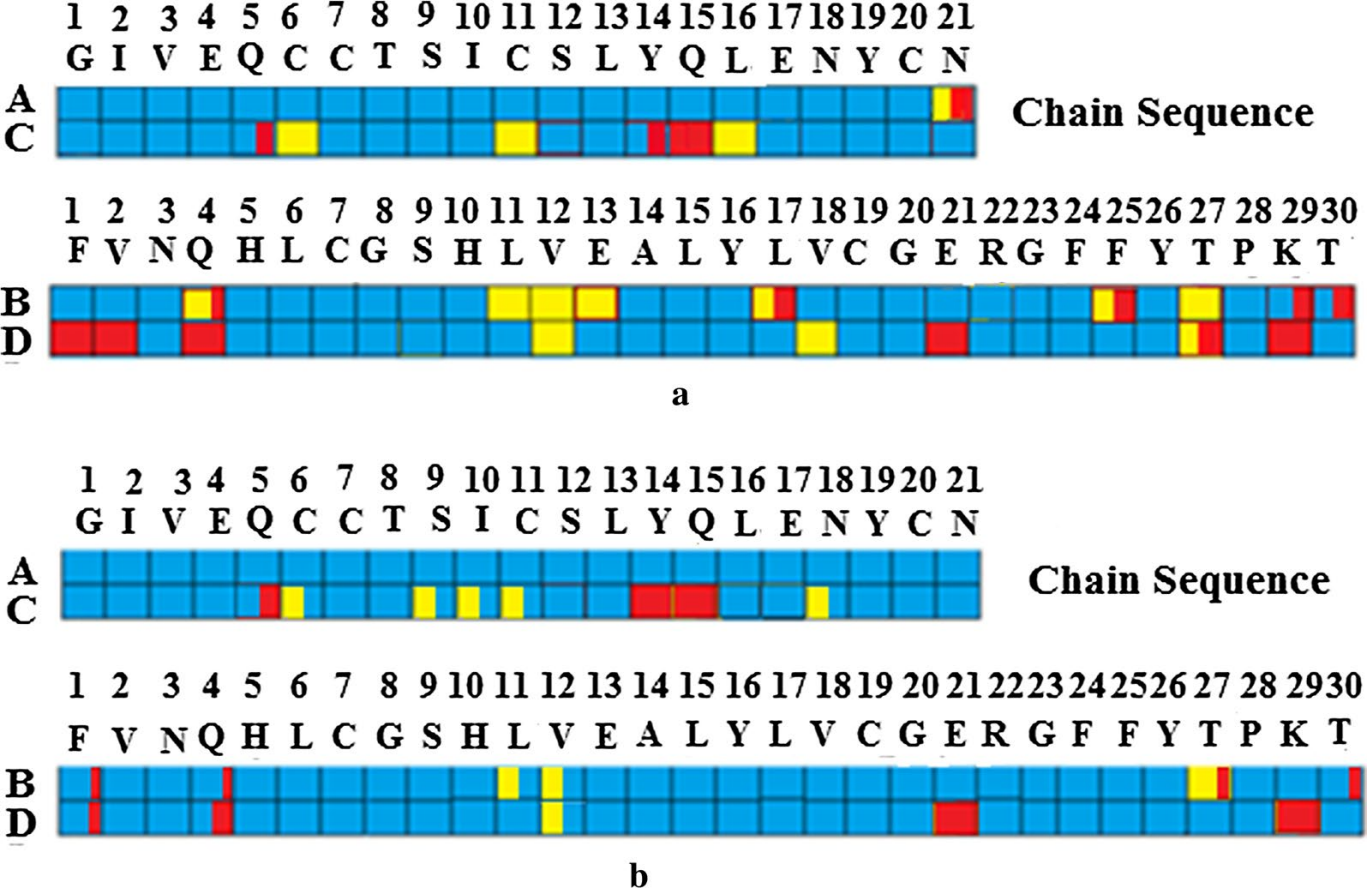
Analysis of the correspondence of amino acid modelling and electron density quality in **a** Insugen (I) and **b** Intergen (II) HR insulins. Colour codes: *blue* excellent quality electron density with minimal problems for modelling a clear single conformation, *orange* clear electron density with two distinct conformations modelled, *red* poorly defined electron density with problems in fitting a meaningful structure, *blue* + *red* single conformation modelled, mainly well-defined but with some minor problems, *orange* + *red* two distinct conformations modelled, mainly well-defined but with some minor problems

Properties of the electron density for Insugen (I) are summarised in colour code in Fig. 11a and in further detail in Additional file 1: Tables S3a–d.

##### Insugen (I) chain A

The electron density of Insugen (I) chain A is of very high quality (mainly blue) with few problems associated with fitting the amino acid residue structures; only the C-terminal residue N21 exhibits a double conformation with two weak regions of density at the end of the chain.

##### Insugen (I) chain C

In contrast chain C exhibits the following characteristics: residues Q5, Y14 and Q15 have mainly good density but with some poorly defined regions; residues C6 and C11 participating in an S–S bridge, and L16 demonstrate clear electron density but corresponding to double residue conformations with good geometry (orange). The remaining residues are clearly defined in strong electron density (blue).

##### Insugen (I) chain B

The electron density of Insugen (I) chain B exhibits the following characteristics: residues L11, V12, and E13 and T27 have clear electron density with two distinct conformations (orange); residues Q4 and L17 show mainly clear double conformations but with some poor density at the extreme end; residue F25 clearly adopts two conformations but both phenyl rings A and B occupy very weak regions of density; residues K29 and T30 are mainly clear single conformations but with some terminal disorder. The remaining residues are clearly defined in strong electron density (blue).

##### Insugen (I) chain D

In contrast the electron density of Insugen (I) chain D can be described as follows: residues F1, V2, Q4, E21 and K29 have overall poorly defined electron density; residues V12 and V18 have clear double conformations (orange); residue T27 is mainly a clear double conformation but with some missing terminal density. The remaining residues are clearly defined in strong electron density (blue).

##### Overall comments on Insugen (I)

For the Insugen (I) structure the following points may be considered.

1. Why is chain A so well ordered while the related chain C shows a number of double conformations and poorly defined residues?
2. Chains B and D both show a number of double conformations. Double conformations L11, E13 and R22 occur only in chain B; the double conformation V18 occurs only in chain D; double conformations V12 and T27 occur in both chains B and D. L17, R22, F25 and T30 are disordered in chain B alone; F1, V2 and Q4 are disordered in chain D alone; E21, T27 and K29 are disordered in both chain B and D.

It may be possible to rationalise these differences for example via molecular dynamics simulations.

##### Implications for the biological activity

The residues most likely to affect biological activity in an adverse way are those which display conformational differences between the corresponding chains A and C, or between chains B and D, particularly with respect to the way the residues have been rated in Fig. 2.

It follows that the most likely residues are by virtue of:

1. being disordered: PheB25, and to a lesser extent GlnC5, AsnA21, LysB29, LysD29 and ThrB30;
2. exhibiting two clear conformations: CysC6–CysC11, LeuB11 and ValB12, and to a lesser extent LeuC16 and GluB13.

**Fig. 12:**
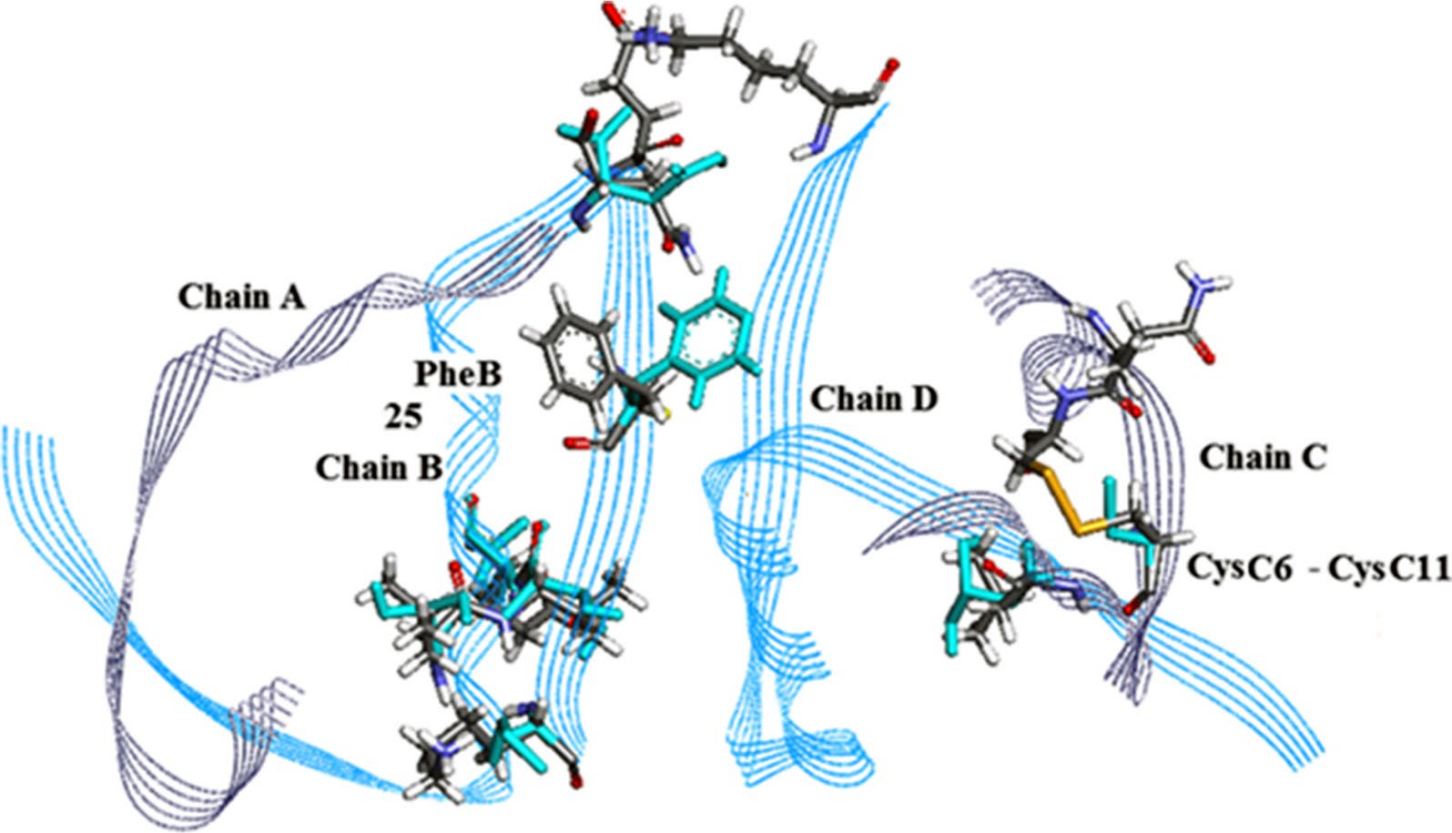
Distribution of residues possibly associated with receptor binding and biological activity of HR insulin, Insugen (I). The major concentration of residues occurs on chain B (*blue*) which includes the residue Phe25B discussed in the definitive account of the porcine insulin X-ray structure by Baker et al. [1]. In the Insugen (I) structure Phe25B occupies two distinct well defined conformations as shown here. It is of interest to note that in Intergen (II) HR insulin Phe25B has a clear well defined conformation. Alternative conformations in Insugen (I) residues are coloured *blue* here. A minor group of residues occurs on chain C (coloured *grey*). Drawn with Accelrys Discovery Studio 3 [23]

The distribution of these residues in the crystal asymmetric unit is shown in Fig. 12. They clearly form two distinctly concentrated groups possibly related to the mode of binding or interaction with the receptor.

#### Peptide side chain electron density and conformations in Intergen (II) [PDB 3W7Y]

Properties of the electron density for Intergen (II) are summarised in Fig. 11b.

##### Intergen (II) chain A

The electron density of Intergen (II) chain A is of very high quality with no major problems associated with fitting the amino acid residue structures and no multiple conformations or other disorder.

##### Intergen (II) chain C

In contrast chain C exhibits the following characteristics: residues 1–4, 7, 8, 12, 13, 16,17,19–21, have clear well defined density; residue Q5 has mainly clear density but with missing terminal density; residues S9, I10, N18 and C6–C11 are modelled as single conformations but are probably well ordered double conformations (all shown in orange in Fig. 11b; Y14 has very poor electron density and is fitted as Ala; Q15 also has very poor density and is disordered).

##### Intergen (II) chain B

Chain B exhibits the following characteristics: residue F1 has mainly clear density but with missing terminal density; residues 2–10, 13–26 and 28–30, have clear well defined density; residues L11 and V12 are modelled as single conformations but are probably well ordered double conformations, whereas residue T27 is in clear well defined density and is modelled as a double conformation but has missing terminal density (all three are shown in orange in Fig. 11b).

##### Intergen (II) chain D

Chain D exhibits the following characteristics: residues 2,3, 5–11, 13–20, 22–28, and 30 are all well defined in clear electron density; F1 is in clear but weak density; Q4 is largely well defined but has missing terminal density; V12 is modelled in a clear single conformation but is in density that strongly suggests it is disordered in two clear conformations (orange in Fig. 11b); E21 and K29 are poorly defined with weak density that does not include all atoms in the residue chains.

##### Overall comments on Intergen (II)

As for the Insugen (I) structure the following points can be made for Intergen (II). Why is chain A so well ordered while chain C shows a number of double conformations and poorly defined residues? Chain B shows one double conformation. There are no double conformations in chain D.

#### Comparison of the Insugen (I) and Intergen (II) structures

Referring to Fig. 11:

1. Both A-chains have mostly well-defined electron density with very few problems in their interpretation.
2. For the C-chains the only notable difference here lies in the assignment of a double conformation for the C6–C11 disulphide bridge in Insugen (I). As mentioned above the electron density for Intergen (II) in this region, Fig. 16, strongly suggests that it might be possible to model a double conformation here as well.
3. Comparison of the B-chains of Insugen (I) and Intergen (II): differences here occur for residues L11, V12 and R22 which have double conformations in Insugen (I) and T27 which has a double conformation in Intergen (II). Insugen (I) chain B also has problem residues E13, L17, E21, F25, T27, K29 and T30, which are well behaved in Intergen (II). Intergen (II) chain B has one residue T27 modelled as a double conformation but which is single in Insugen (I).

There are a number of differences between Insugen (I) chain D and Intergen (II) chain D. In Insugen (I) F1, V2 and T27 all have problem electron density but are well behaved in Intergen (II); Q4, E21 and K29 have weak or poorly defined electron density in both structures; residues S9, V12 and V18 have double conformations in Insugen (I) but not in Intergen (II).

#### General comments on Insugen (I) and Intergen (II) structures

The above analysis has indicated that in both the Insugen (I) and Intergen (II) structures the sequence equivalent protein chains A and C, and B and D, respectively exhibit significant differences with respect to their corresponding amino acids such as double conformations and quality of the electron density. It is of interest to note that Baker et al. [1] in discussing the 1.5 Å X-ray structure of porcine insulin, report the presence of seven disordered amino acid residues: two in chain B (ArgB22 and LysB29) and five in chain D (GlnD4, ValD12, GluD21, ArgD22 and ThrD27). Of these only two amino acids in Insugen (I) ArgB22 and ValD12, have double conformations. The question of double conformations and poorly defined or absent electron density in the recombinant human insulin structures and the widespread lack of correspondence between the two raises two questions: (1) what is the origin of these differences? And (2) do they affect the therapeutic properties of these preparations? With respect to question (1) the possibilities include (a) method of preparation including folding of the recombinant amino acid-chains and (b) the forces in play when the crystal is cryo cooled prior to X-ray data collection. With respect to question (2) it is well known that differences in the form of a therapeutic insulin preparation with respect to the naturally occurring insulin can induce the production of antibodies in patients. No such indication has been noted with respect to the widespread use of either Insugen (I) or Intergen (II) but is nevertheless a possibility which should be borne in mind.

### Some further selected details

#### Insugen (I) structure: chain C(3) S–S bridge between Sγ6–Sγ11

**Fig. 13:**
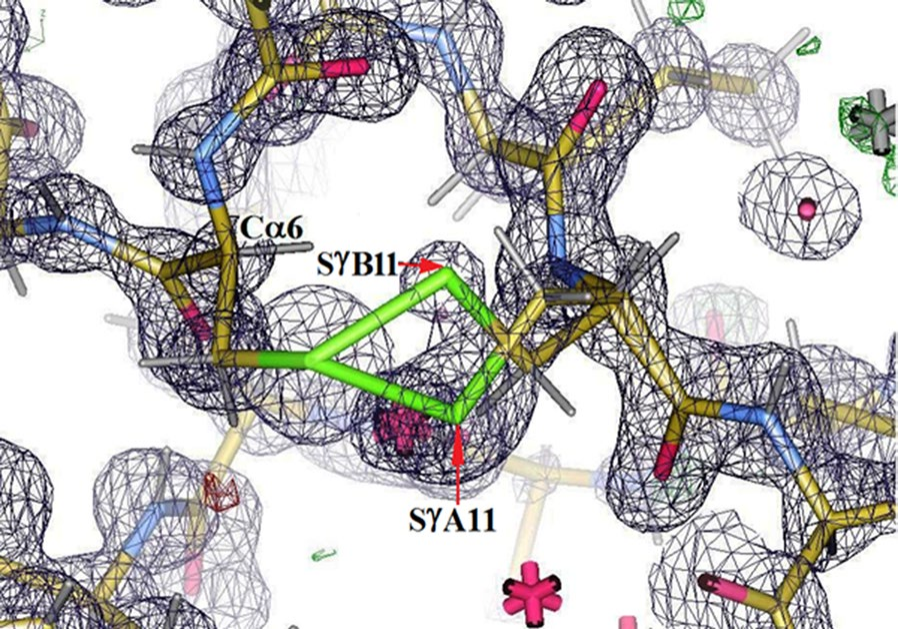
Electron density in the disordered internal S–S bridge in Insugen (I) chain C between Sγ6–Sγ11. Atom Sγ11 occupies two clear sites A (80%) and B (20%). Sγ6 occupies a single site. Drawn with WinCoot 0.3 [19]

The electron density excerpt below, Fig. 13, reveals the distinct disordering in this region. This shows the electron density in the disordered internal S–S bridge of Insugen (I) chain C(3) between Sγ6–Sγ11. Atom Sγ11 occupies two clear sites A (80%) and B (20%). Sγ6 occupies a single site.

**Fig. 14:**
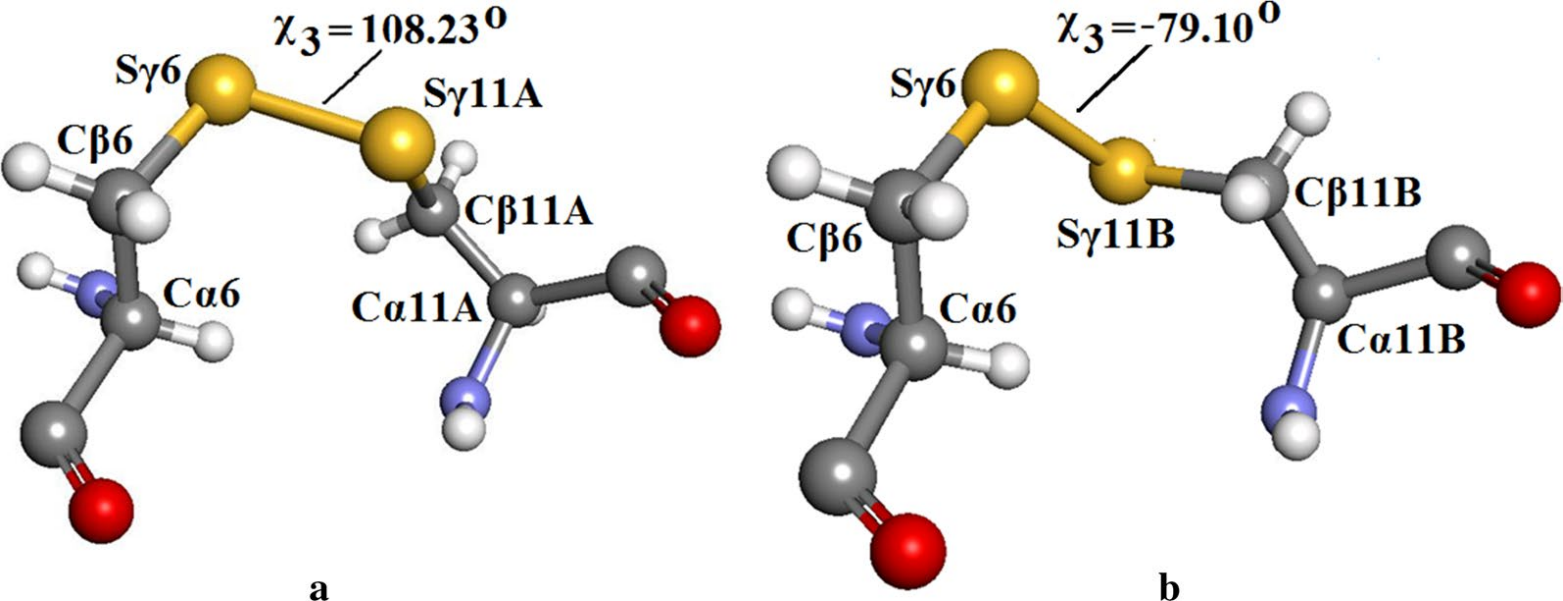
**a** Insugen (I) structure: chain C(3) S–S bridge between Sγ6 and Sγ11 showing the geometry of the *major* conformation part A of the disordered S–S bridge in chain C(3). **b** Insugen (I) structure: chain C(3) S–S bridge between Sγ6 and Sγ11 showing the geometry of the *minor* conformation part B of the disordered S–S bridge in chain C(3). Drawn with Accelrys Discovery Studio 3 [23]

Figure 14a shows details of the Insugen (I) structure: chain C(3) S–S bridge between Sγ6–Sγ11 showing the geometry of part A of the disordered S–S bridge in chain C(3). For Sγ6–Sγ11A: Cα6–Cβ6 = 1.532 Å, Cβ6–Sγ6 = 1.815 Å, Sγ6–Sγ11A = 2.119 Å, Sγ11A–Cβ11A = 1.785 Å, Cβ11A–Cα11A = 1.547 Å; Cα6–Cβ6–Sγ6 = 114.16°, Cβ6–Sγ6–Sγ11A = 98.05°, Sγ6–Sγ11A–Cβ11A = 104.10°, Sγ11A–Cβ11A–Cα11A = 112.22°; torsion angle χ^3^ = Cβ6–Sγ6–Sγ11A–Cβ11A = 108.23° corresponds to right handed chirality [26].

Figure 14b shows the geometry of part B of the disordered S–S bridge in chain C(3) for Sγ6–Sγ11B: Cα6–Cβ6 = 1.532 Å, Cβ6–Sγ6 = 1.815 Å, Sγ6–Sγ11B = 1.966 Å, Sγ11B–Cβ11B = 1.771 Å, Cβ11B–Cα11B = 1.534 Å; Cα6–Cβ6–Sγ6 = 114.16°, Cβ6–Sγ6–Sγ11B = 112.79°, Sγ6–Sγ11B–Cβ11B = 103.44°, Sγ11A–Cβ11A–Cα11A = 114.26°; torsion angle χ3 = Cβ6–Sγ6–Sγ11B–Cβ11B = −79.10° corresponds to *left handed* chirality [26]. Additional file 1: Figure S7 shows views of the major and minor conformations of this S–S bridge with respect to the secondary structure of the protein.

**Fig. 15:**
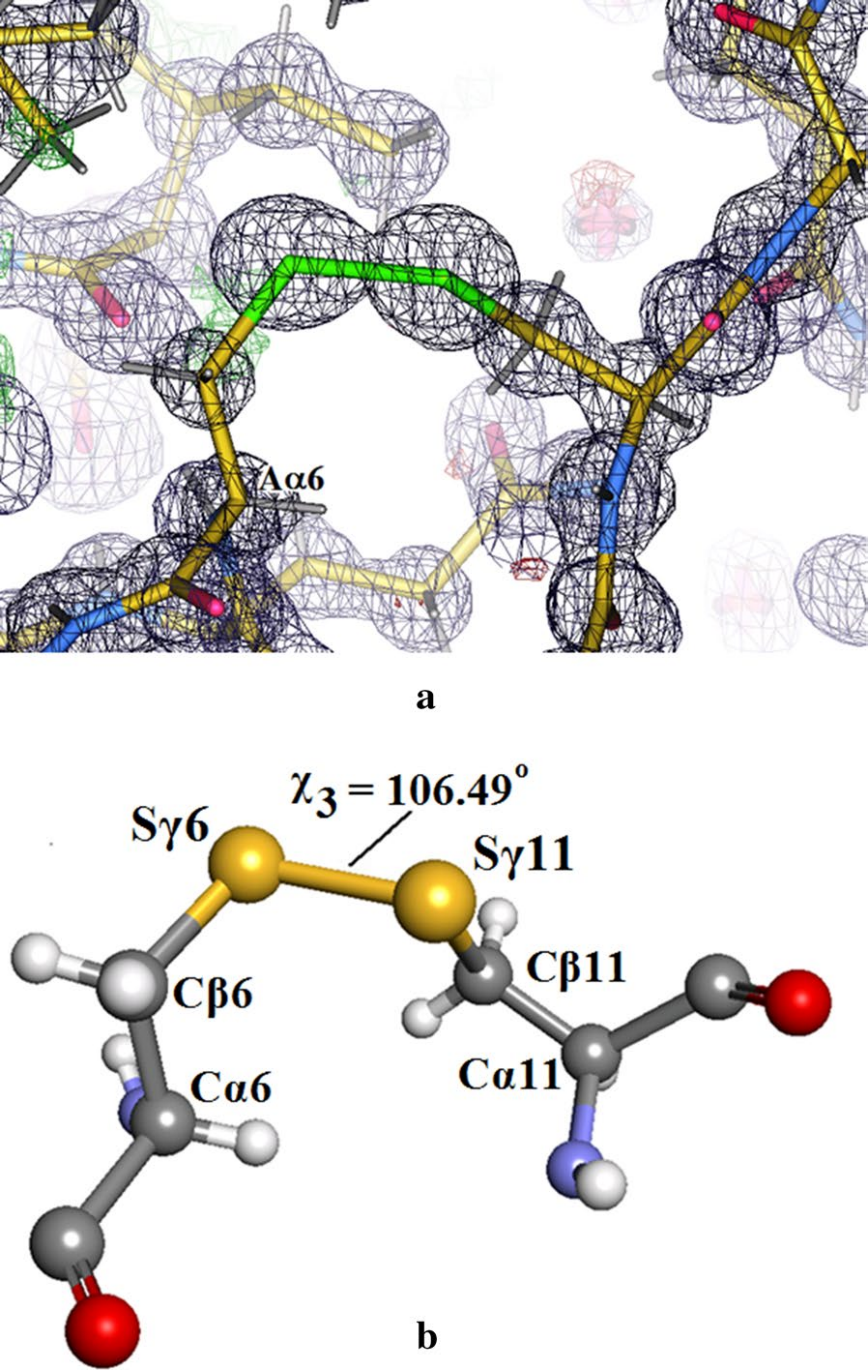
**a** Electron density in the ordered internal S–S bridge Insugen (I) chain A(1) between Sγ6 and Sγ11. Drawn with WinCoot 0.7 [19]. **b** Insugen (I) structure: chain A(1) S–S bridge between Sγ6 and Sγ11 showing the geometry of the ordered S–S bridge after refinement. Drawn with Accelrys Discovery Studio 3 [23]

**Fig. 16:**
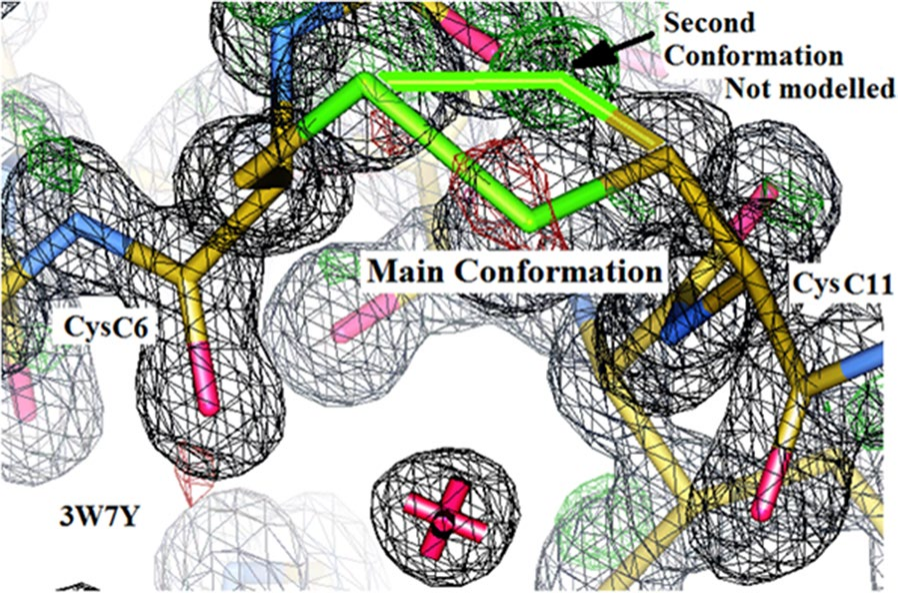
Electron density in Intergen (II) (3W7Y) in the vicinity of the disulphide bridge in chain C CysC6–CysC11. The presence of green density (*arrowed*) suggests the existence of a second conformation, as in the Insugen (I) structure. This second, minor, conformation has not been modelled in deposited Intergen (II) structure. Drawn with WinCoot 0.7 [19]

Figure 15a shows the electron density in the ordered internal S–S bridge in chain A(1) between Sγ6–Sγ11. Compare with Fig. 13 which shows the corresponding S–S bridge in chain C(3) in which atom Sγ11 is disordered into two sites A (80%) and B (20%) and Fig. 16 which shows the electron density in this region in Intergen (II) which has been modelled and refined as a single ordered cysteine but appears, in fact, to be an ordered double conformation as in Intergen (I).

Figure 15b shows the geometry of the ordered internal S–S bridge in Insugen (I) chain A(1) Sγ6–Sγ11: Cα6–Cβ6 = 1.532 Å, Cβ6–Sγ6 = 1.797 Å, Sγ6–Sγ11 = 2.051 Å, Sγ11–Cβ11 = 1.806 Å, Cβ11–Cα11 = 1.533 Å; Cα6–Cβ6–Sγ6 = 116.37°, Cβ6–Sγ6–Sγ11 = 103.66°, Sγ6–Sγ11–Cβ11 = 101.86°, Sγ11A–Cβ11A–Cα11A = 113.83°; torsion angle χ = Cβ6–Sγ6–Sγ11B–Cβ11B = 106.49° is close to the value of χ = 108.23° in the major conformation in chain C(3) and again corresponds to *right handed* chirality [26] the minor conformation being *left handed*, χ3 = −79.10°.

### Conclusions on the comparison between Insugen (I) and Intergen (II) structures

Possible explanations for the observed bifurcation of chain C(3) Sγ6–Sγ11 disulphide are as follows: CysC6 is hydrogen bonded to a water molecule and there are several other waters modelled in this region which may be associated with greater conformational flexibility compared to CysA6. In addition CysA6 is in a hydrophobic pocket devoid of solvate molecules and consequently the disulphide may be more restricted by this environment. This is supported by the fact that the section of chain D close to chain A(1) Sγ6–Sγ11 disulphide is disordered (residues D1, 2 and 4) whereas the section of chain B close to chain C(3) Sγ6–Sγ11 disulphide is not, Fig. 11a and Additional file 1: Tables S3c, d. In “Molecular dynamics” the results of a molecular dynamics study of this observed order/disorder in the Sγ6–Sγ11 disulphides are presented.

#### Intergen (II) structure: chain C(3) S–S bridge between Sγ6–Sγ11

Inspection of the deposited X-ray structure of Intergen (II) (3W7Y), indicates that no attempt was made to model CysC6–CysC11 in chain C in a double conformation. However the superposition of the refined Insugen (I) chain C with the 3W7Y chain C indicates that the alternate conformation of the disulphide from residues CysC6–CysC11 is likely also to be present, but not modelled, in the 3W7Y structure. This is indicated by the presence of negative electron density (green) in the same position as the CysC11 γ sulphur atom in the second (minor) conformation and positive (red) electron density in the over modelled main conformation, Fig. 16.

The possibility of this effect being accounted for by radiation damage in the Insugen (I) structure was investigated by closely inspecting the intensity data collected. This led to the conclusion that there is no global suggestion of radiation damage in the data. Next a number of subsets of data were integrated and scaled and the minimum set of data with acceptable completeness was assembled by using images 1–600 (the first third of the data). When solved and initially refined there was still evidence for the second conformation at this disulphide bond. As two clear conformations, rather than complete disorder have been assigned successfully it may be concluded that this is a reflection of the true state in the crystal, rather than radiation damage. Further examination of the difference in the disulphides A6–A11 (single ordered conformation) and C6–C11 (clear ordered double conformation) may be explained by the difference in solvent exposure. C6 is less than 4 Å from the nearest solvent molecule and there are several waters modelled in that area which may give greater conformational flexibility to the region. A6 is in a hydrophobic pocket and consequently the disulphide may be more restricted by that environment. This is supported both by the fact that the section of chain D in this vicinity of the part of the molecule is also disordered (see above).

#### Solvated propanol in Insugen (I)

**Fig. 17:**
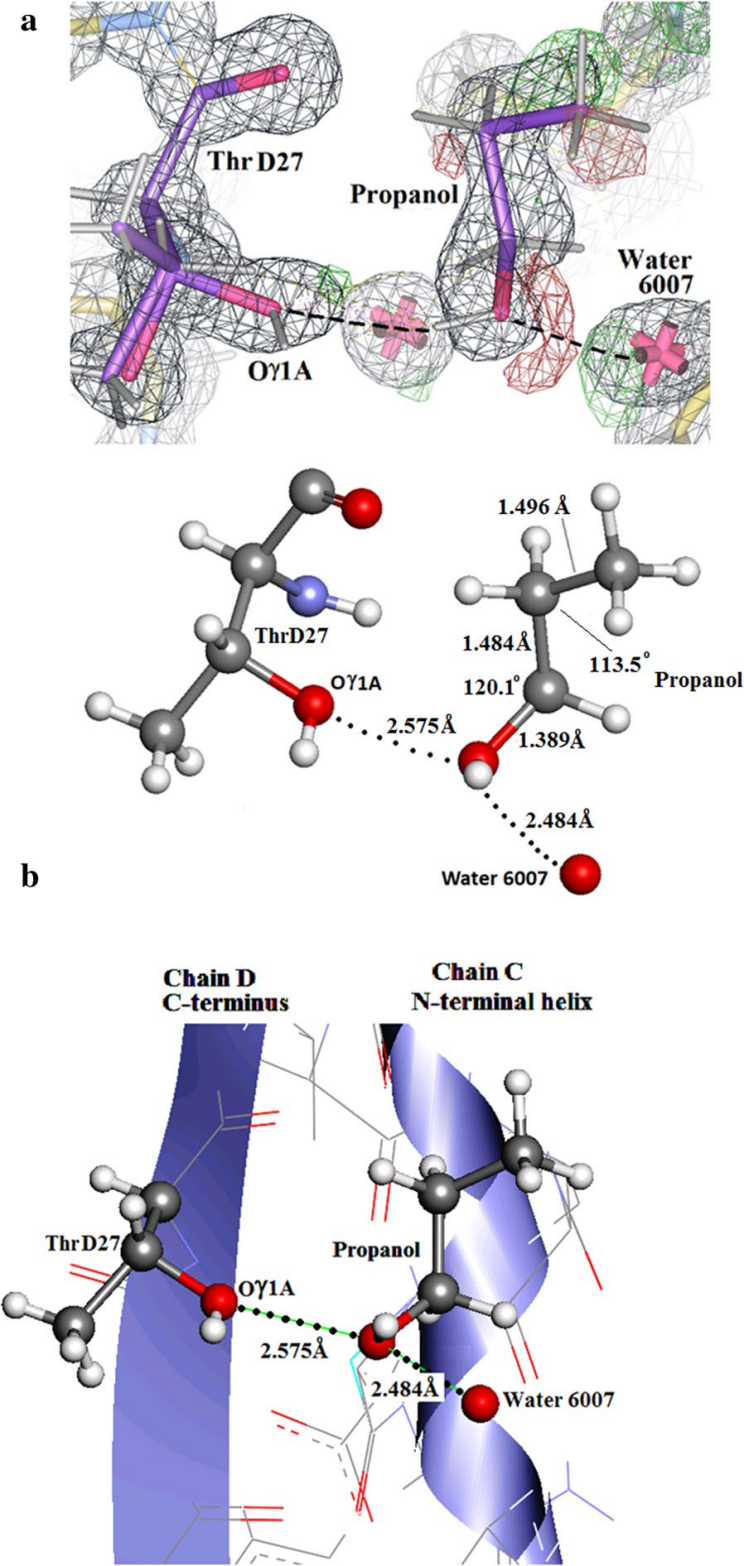
**a** Insugen (I) COOT electron density in the vicinity of ThrD27. Electron density for the solvated propanol 5001 and water 6007 are shown. H-bonds between propanol and Oγ1A of ThrD27 and water 6007, respectively are indicated. Drawn with WinCoot 0.7 [19]. **b** Detail produced by Discovery Studio 3 (Accelerys) [23, 35] showing the solvated propanol in Insugen (I) with respect to OG1A ThrD 27 and water 6007. **c** The solvated propanol in Insugen (I) with respect to Oγ1A Thr D27 and water 6007. Drawn with Discovery Studio 3 (Accelerys) [23, 35]

The ultra-high resolution Insugen (I) X-ray structure has been found to include an unexpected solvated propanol molecule (POL5001), Fig. 17a. This solvate forms H-bonds with the prominent Oγ1A of ThrD27 in chain D(4), water 6002 and water 6007. The electron density for this solvate is clear (Fig. 17a) and the geometry of the refined propanol is excellent (Fig. 17b). ThrD27 in the Insugen (I) structure is cleanly split into two parts A and B as can be seen in Fig. 17a. To the best of our knowledge no other insulin structure has been shown to include structurally ordered propanol.

Figure 17c shows the propanol molecule in Insugen (I) lying in a binding pocket formed by rigid body movement of the first helix of chain C with respect to the structure of Baker et al. [1] (PDB 4INS). Interestingly, there is a similar movement of chain A in spite of there being no propanol solvate in this region. The C-terminal of chain D is also displaced towards the propanol binding pocket, while on chain B the movement is in the opposite direction.

**Fig. 18:**
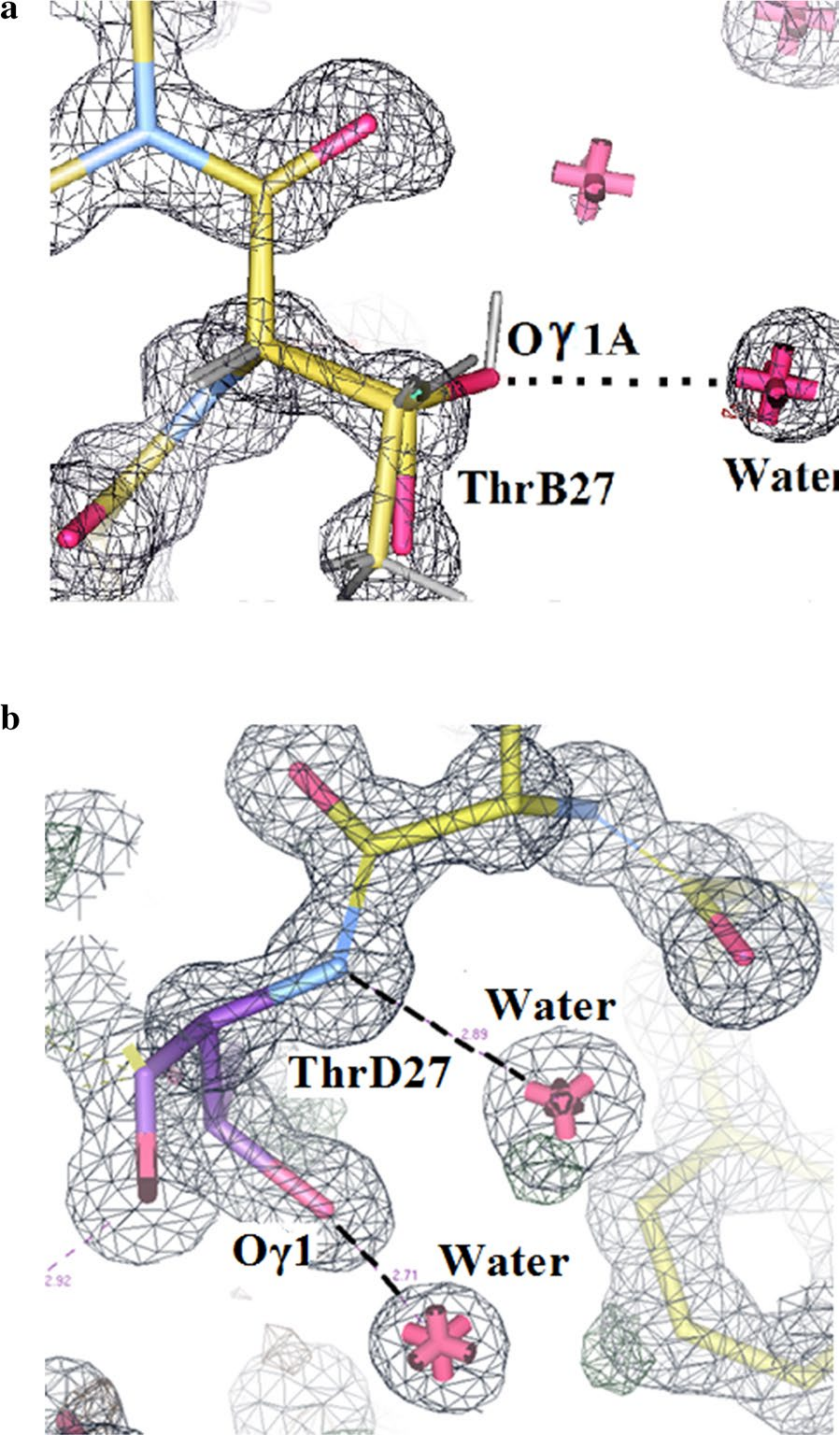
**a** Insugen (I) HR insulin: WINCOOT 0.7 [19] electron density in the vicinity of ThrB27 in chain B. Unlike the corresponding site ThrD27 in chain D there is no evidence of solvated propanol in this site. **b** Intergen (II) 3W7Y: WINCOOT 0.7 [19] electron density in the vicinity of Thr D27. There is no evidence of solvated propanol in this site. The same applies to the Intergen (II) ThrB27 site

Figure 18a shows the electron density in Insugen (I) in the vicinity of ThrB27 in chain B. There is no evidence of solvated propanol bound in this site. Similarly Fig. 18b shows Intergen (II) in the vicinity of chain D ThrD27 again with no propanol present, as is also the case for Intergen (II) chain B ThrB27.

#### Comments on the solvated propanol in Insugen (I)

It is interesting to note that Step 12 of US Patent Application Number US 13/032,797 [27] describes the use of n-propanol in a process for producing improved preparations and methods for manufacturing substantially liquid preparations of RH insulin API. It is possible that the manufacture of Insugen (I) has included a similar step and this is the origin of the bound propanol revealed in the ultra-high resolution X-ray structure described here. It is possible that the presence of propanol in this insulin preparation may have consequences with respect to its biological/therapeutic characteristics [28]. Further evidence for the assignment of propanol in this pocket of electron density was obtained by modelling in a number of different likely possibilities. Of these propanol emerged as the most likely candidate (see Additional file 1: Text S2, Figure S4).

The use of the molecular modelling procedures described in “Molecular dynamics” to investigate reasons for the presence of propanol in the binding site located on chain D of Insugen (I) described here is currently in progress.

#### The Zn sites in molecules 1 and 2

Insugen (I) and Intergen (II) have been synthesised to include the Zn ions present in naturally occurring insulins. The Zn ions are an essential feature in the formation of the crystal structure and are located on a crystallographic three-fold axis. In porcine insulin 2 Zn crystals [1], three insulin dimers are assembled around two zinc ions, 15.82 Å apart on the threefold axis. Each zinc is coordinated to three symmetry related Nε atoms of residue His10B, both at 2.05 Å, and to three water molecules at 2.36 and 2.21 Å, in molecules 1 (chains A and B) and 2 (chains C and D), respectively. During the course of the X-ray analysis of Insugen (I) the Zn sites in molecules 1 and 2 were carefully examined.

##### The Zn site in Insugen (I) molecule 1

**Fig. 19:**
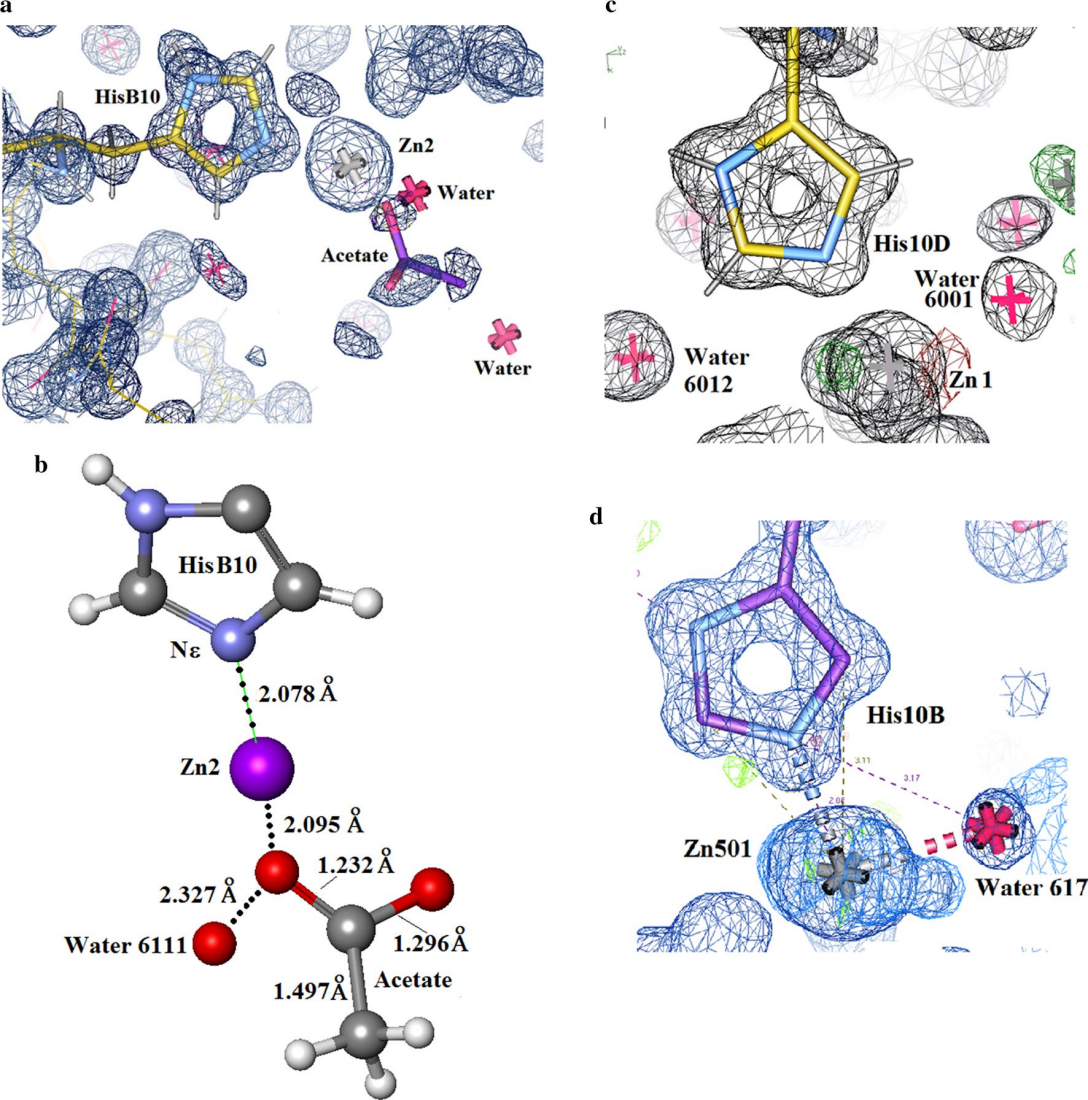
**a** Insugen (I) electron density in the vicinity of Zn2100 (Zn2) in molecule 1: an acetate molecule acetate 2101 has been modelled in this site close to HisB10 in chain B. Drawn with WinCoot 0.7 [19]. **b** The Zn site in molecule 1 of Insugen (I). Zn2 is coordinated to His B10 Nε in chain B as usually observed in insulin structures (e.g Baker et al. [1]) and unexpectedly to a highly ordered acetate molecule acetate2101. Drawn with Accelrys Discovery Studio 3 [23]. **c** Insugen (I) structure electron density showing the vicinity of Zn4100 (Zn1) and HisD10 in chain D. Unlike chain B there is no acetate in this site. Two water molecules have been located whose equivalents are not present in the vicinity of Zn2100 (Zn2) which has the substituted acetate2101. Drawn with WinCoot 0.7 [19]. **d** Intergen (II) electron density in the vicinity of Zn501 in molecule 1 B-chain. Both HisB10 Nε and water 617 coordinate Zn501. Water617 is the only coordinating water. There is no acetate molecule in this site. The same applies to site D. Drawn with WinCoot 0.7 [19]

The electron density in the vicinity of Zn2 in molecule 1 is shown in Fig. 19a. This reveals an unexpected feature which was modelled and successfully refined as a solvated acetate molecule, acetate 2101. Zn2100 is coordinated to both His 2010 Nε in chain B and an oxygen atom of acetate2101 (Fig. 19b). The geometry of acetate2101 (Fig. 19b) and its refined parameters are of excellent quality. Note: the complete coordination sphere around the zinc ion is generated by application of the crystallographic three-fold symmetry.

##### The Zn site in Insugen (I) molecule 2

Figure 19c shows the electron density in the vicinity of Zn1 in Insugen (I) molecule 2. This shows Zn4100 coordinated to His 4010 Nε and two water molecules. This is the normal mode of Zn binding in insulins [1]. Note: as for molecule 1 the complete coordination sphere around the zinc ion is generated by application of the crystallographic three-fold symmetry.

Additional file 1: Figure S8 shows the arrangement of the Zn sites in Insugen (I) with respect to peptide chains B and D and the propanol associated with chain D ThrD27.

##### The Zn sites in Intergen (II) molecules 1 and 2

Figure 19d shows the electron density in the vicinity of Zn501 in the Intergen (II) structure molecule 1. Zn501 is coordinated by His10B Nε as usual and a single water molecule water 617. There is no other solvate in this site. The Zn site in Intergen (II) molecule 2 is structured in the same way. Note: as previously stated the complete coordination sphere around the zinc ion is generated by application of the crystallographic three-fold symmetry.

## Molecular dynamics

### Insugen (I)

#### Introduction

As discussed previously in the ultra-high resolution X-ray structure of Insugen (I) in the internal disulphide bridge of chain C (CysC6–CysC11) CysC11 is disordered into two sites: A (80%) and B (20%), Figs. 13, 14a, b. However the corresponding disulphide bridge in chain A is not disordered, Fig. 15a, b. In this section molecular dynamics calculations have been employed in order to investigate and find a rational explanation for this difference.

#### Materials and methods

In order to prepare for the molecular dynamics (MD) simulations, two pdb files, Cys11_80percent.pdb and Cys11_20percent.pdb were generated from the original high resolution crystal structure by editing the atom records for Cys11, generating two separate pdb files, one with the chain C CysC6–CysC11 disulphide in the major (80%) conformation, the other with the minor (20%) conformation. Following this, both structures, including water molecules in the crystal structure were subjected to energy minimisation using HyperChem 8 Professional^(^™^)^ [29]. Energy minimisation was performed using the AMBER3 force field [30], using the Polak-Ribere conjugation gradient [31], with only the original contents of the crystal structure contained in a periodic box, since the object of the MD simulation was to explain the disorder in the original unit cell of the high resolution crystal structure, rather than a protein under normal solvated biological conditions.

As described below MD simulations were then performed. Two MD simulations were run for each pdb file. The first simulation was run at 310 K for 300 ps, using an initial heat time of 5 ps, with data collected every 0.01 picoseconds, with a time step of 0.002 ps, using NVT dynamics with a Berendsen thermostat [32]. The second simulation was run at a higher temperature of 320 K, an initial heat time of 2.5 ps. Data collection and time steps remained the same as the first simulation. The MD simulations were carried out using the leapfrog algorithm [33], with AMBER3 [30] being used as before. Data was collected with respect to torsion angles for Cys6–Cys11 S–S bonds from both chains A and C, along with root mean square deviations for the torsion angles. RMSD values at the end of the simulations were also collected for both insulin molecules in the crystal asymmetric unit (Table 4a, b).

**Table 4:** RSMD values (Å) for HR insulin structures after the MD simulation

	Molecule 1 (chain A & B)	Molecule 2 (chain C & D)
(a) Insugen (I)		
310 K		
Major	1.407	1.351
Minor	1.794	1.987
320 K		
Major	1.739	1.639
Minor	2.003	2.155
(b) Intergen (II)		
310 K	1.501	2.125
320 K	1.505	2.519

#### Results

**Fig. 20:**
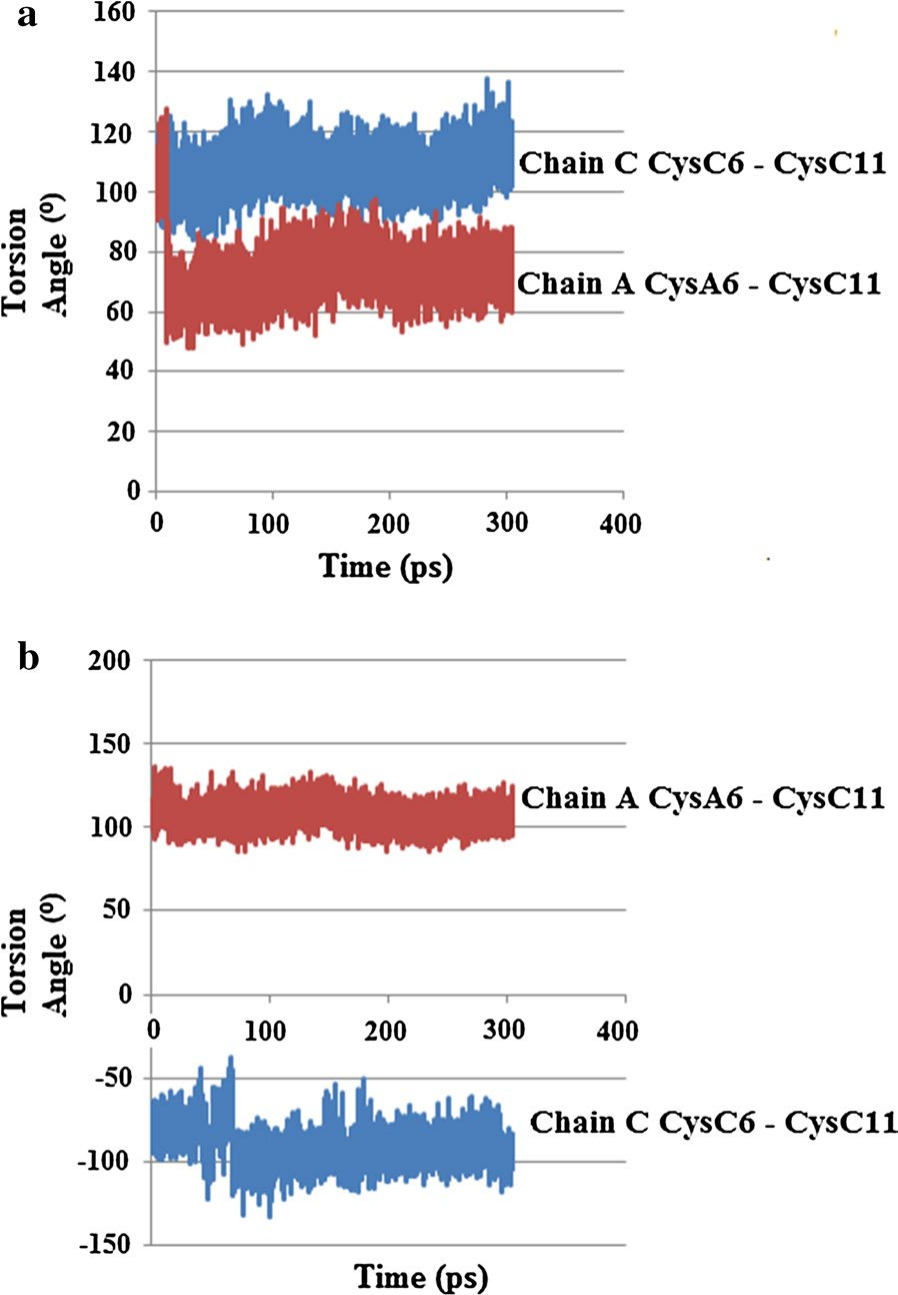
**a** Plot of torsion angle χ3 changes for Cys6–Cys11 showing changes for the *major* conformation of HR insulin, Insugen (I) for the MD simulation carried out at 310 K. **b** Plot of torsion angle χ3 changes for Cys6–Cys11 showing changes for the *minor* conformation of HR insulin, Insugen (I) for the MD simulation carried out at 310 K

**Fig. 21:**
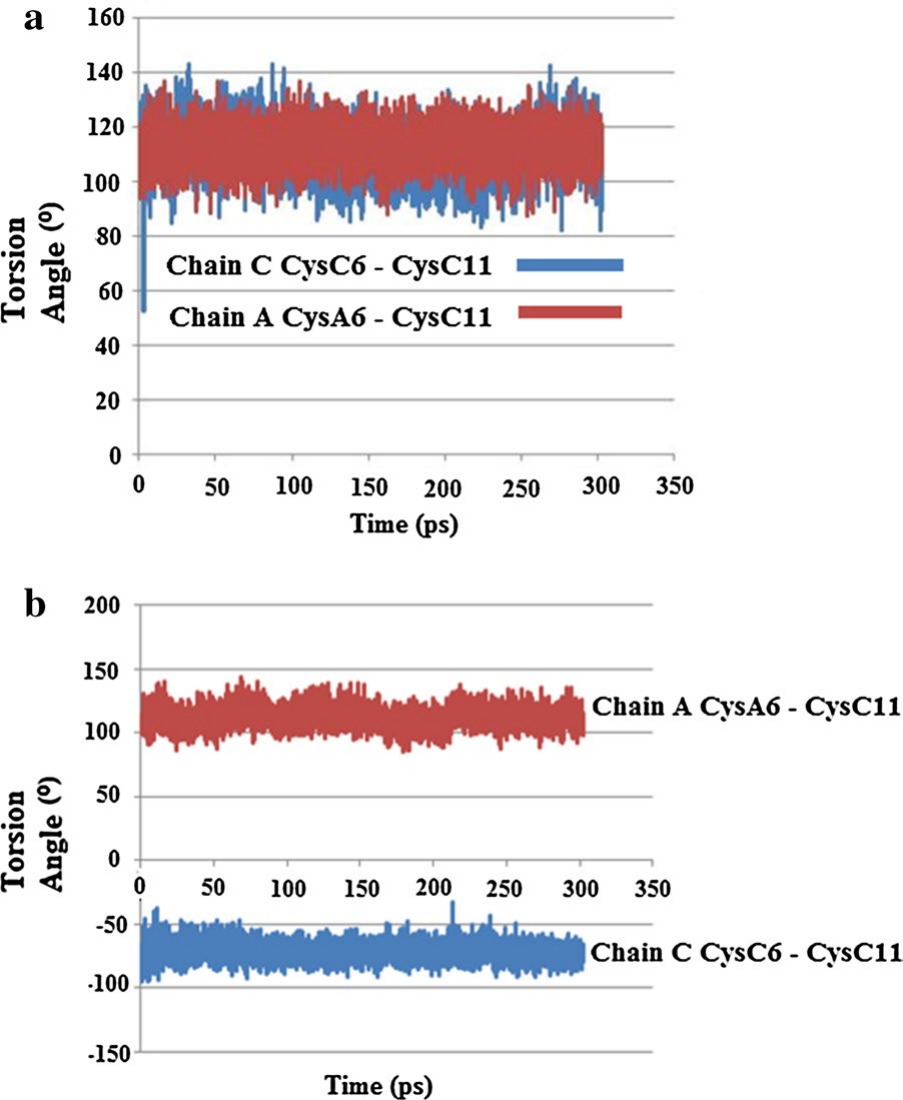
**a** Plot of torsion angle χ3 changes for Cys6–Cys11 for the *major* conformation in HR insulin, Insugen (I) for the MD simulation carried out at 320 K. **b** Plot of torsion angle χ3 changes for Cys6–Cys11 for the *minor* conformation in HR insulin Insugen (I) for the MD simulation carried out at 320 K

**Fig. 22:**
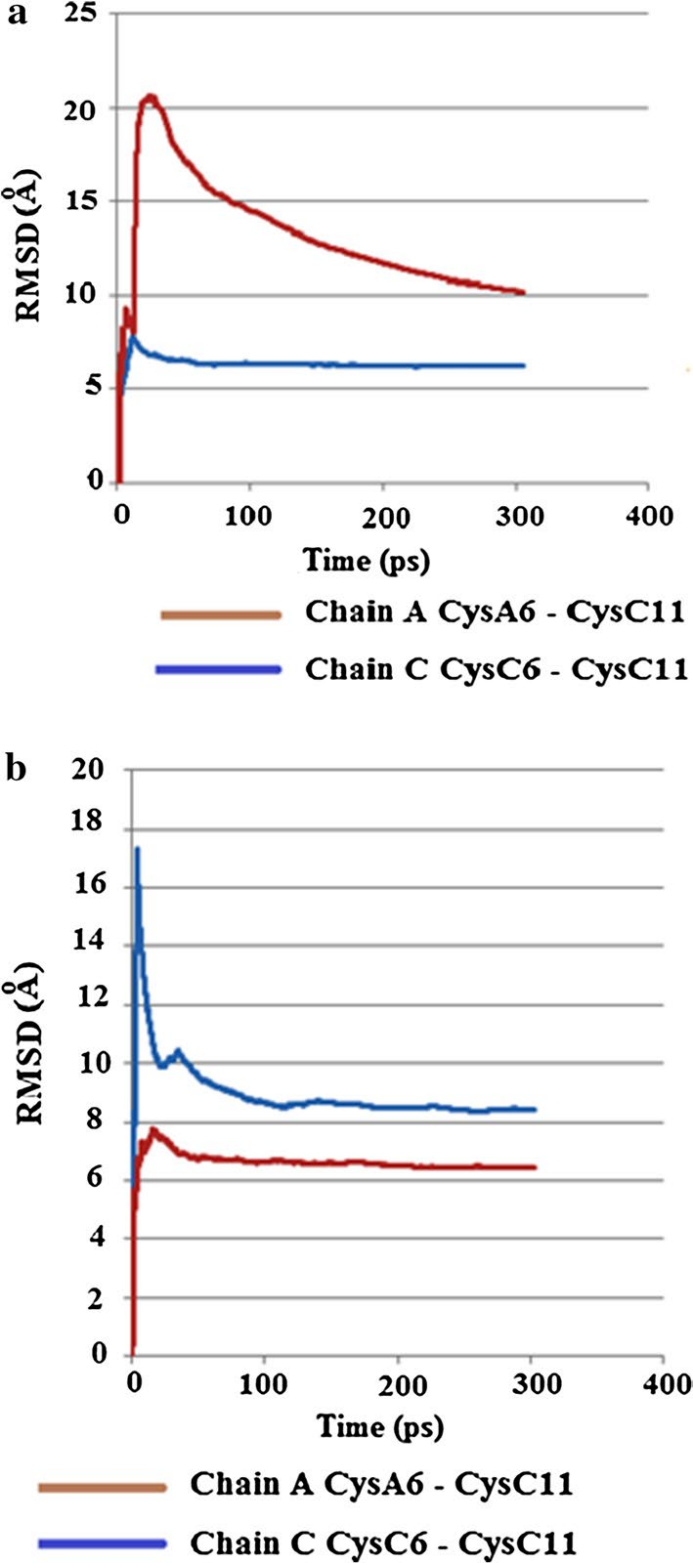
**a** Plot of RMSD kinetics of Cys6–Cys11 torsion angles χ3 for the HR insulin, Insugen (I) MD simulations carried out on the major conformation of chain C Cys6–Cys11 at 310 K. **b** Plot of RMSD kinetics of Cys6–Cys11 torsion angles χ3 for the HR insulin Insugen (I) MD simulations carried out on the major conformation of chain C Cys6–Cys11 at 320 K

**Fig. 23:**
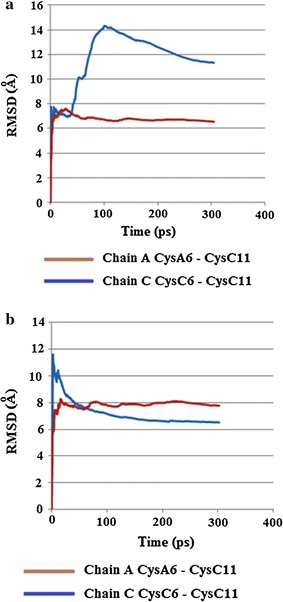
**a** Plot of RMSD kinetics of Cys6–Cys11 torsion angles χ3 for the HR insulin, Insugen (I) MD simulations carried out on the minor conformation of chain C Cys6–Cys11 at 310 K. **b** Plot of RMSD kinetics of Cys6–Cys11 torsion angles χ3 for the HR insulin Insugen (I) MD simulations carried out on the minor conformation of chain C Cys6–Cys11 at 320 K

The results of both simulations showed several notable changes regarding torsion angle χ3 of the internal Cys6–Cys11 disulphide bonds, where χ3 is defined by the atoms Cβ6–Sγ6–Sγ11–Cβ11. At the lower temperature (310 K) in the major conformation CysA6–CysA11 of chain A underwent a conformation change around 8 ps, decreasing from about 120° to 50°, and then increased marginally before staying relatively constant between 60° and 80°. Chain C stayed relatively constant between 90° and 120°, Fig. 20a. The minor conformation for CysC6–CysC11 of chain A stayed relatively constant between 100° and 130°. CysC6–CysC11 of chain C, however, showed several changes. At about 36–41 ps there is an increase in the torsion angle χ3, followed by a decrease (41–47 ps), then another increase (41–66 ps), then another decrease before χ3 remains relatively steady for the rest of the simulation at roughly −80° to −100°, Fig. 20b. For the simulations at 320 K, the most noticeable change for the major conformation of chain C showed a very sharp, but transient decrease to around 50°, between approx 2–4 ps, before increasing and remaining relatively constant for the rest of the stimulation, while chain A remained relatively constant between 100° and 120°, Fig. 21a. Both Cys6–Cys11 of chains A and C stayed relatively constant for the minor conformation, Fig. 21b. Examination of the RMSD torsion angle kinetics for both the major and minor conformations of Cys6–Cys11 for the MD simulations show that for the major conformation at 310 K, RMSD values are much higher for CysA6–CysA11 of chain A, Fig. 22a. However for the minor conformation at 310 K and the major conformation at 320 K, the RMSD values are much higher for CysC6–CysC11 in chain C for all or the majority of the simulation, Figs. 22b and 23a. For the minor conformation at 320 K, RMSD values start higher for Cys C, but then fall below Cys A after 60 ps, Fig. 23b.

### Intergen (II) 3W7Y

#### Materials and methods

The structure of Intergen (II) was also minimised using the method described above for Insugen (I), but was not split into two pdb files beforehand as no disordering was modelled for this structure, (Fig. 16). Following this, MD simulations were run at 310 K and 320 K, again using the methods described above for Insugen (I).

#### Results

**Fig. 24:**
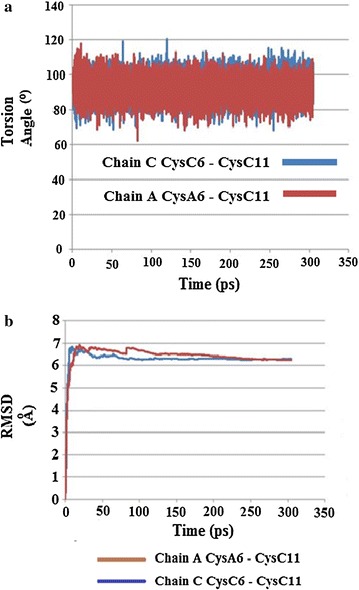
**a** Plot of torsion angle χ3 changes for Cys6–Cys11 in chains A and C in HR insulin Intergen (II) 3W7Y for the MD simulation carried out at 310 K. **b** Plot of RMSD kinetics of Cys6–Cys11 torsion angles χ3 for the HR insulin Intergen (II) 3W7Y in chains A and C, respectively. MD simulations carried out at 310 K

The results for the simulations run at 310 K showed torsion angles χ3 for both Cys 6–Cys 11 in chains A and C largely remaining relatively steady around 80°–100°, except for some transient spikes above or below this range (Fig. 24a).

**Fig. 25:**
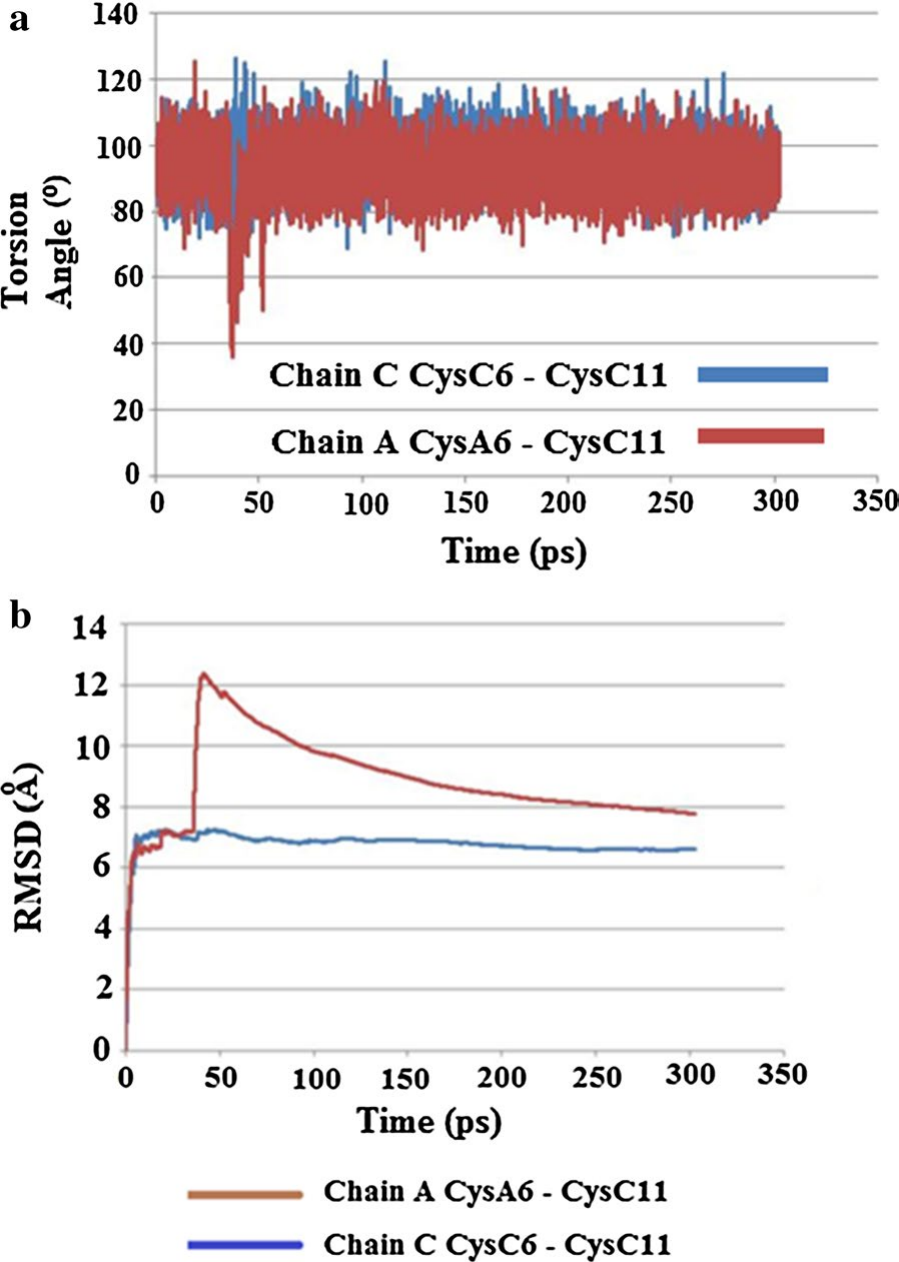
**a** Plot of torsion angle χ3 changes for Cys6–Cys11 in chains A and C in HR insulin Intergen (II) 3W7Y for the MD simulation carried out at 320 K. **b** Plot of RMSD kinetics of Cys6–Cys11 torsion angles χ3 for the HR insulin Intergen (II) 3W7Y in chains A and C, respectively. MD simulations carried out at 320 K

Upon repeating the simulations at the higher temperature of 320 K, torsion angle χ3 for CysA6–Cysa11 in chain A started off mostly steady around 80°–100° up to about 35–55 ps, and then temporarily sharply decreased before increasing again and then remaining relatively constant and mostly steady around 80°–100° for the rest of the stimulation, except for some transient spikes. CysC6–CysC11 of chain C did not show any noticeable changes at the higher temperature, and remained relatively constant around 80°–100°, except for some transient spikes, which is similar to the result obtained for 310 K (Fig. 24b). The RMSD torsion angle kinetics support these changes, showing little difference between Cys 6–Cys 11 in chains A and C over the course of the simulation run at 310 K, but are much higher for Cys 6–Cys 11 in chain A than for Cys 6–11 in chain C for the simulation run at 320 K (Fig. 25a, b).

### Conclusions

From the results of both the torsion angle plots and the RMSD kinetics for the Cys6–Cys11 SS bonds for Insugen (I), it is clear that both the Cys6–Cys11 internal disulphide bridges in chains A and C possess flexibility. However the flexibility of Cys6–Cys11 of chain C appears to be much greater, as both the MD simulations for the minor conformation at 310 K and the major conformation show times when the torsion angle of Cys6–Cys11 of chain C shows rapid decreases followed by rapid increases. In contrast, Cys6–Cys11 of chain A only showed one major change, in the major conformation at 310 K, at all other times staying relatively constant. The rapid changes in torsion angles shown by Cys6–Cys11 of chain C of Insugen (I) would appear to explain why it shows disorder in the original crystal structure.

**Fig. 26:**
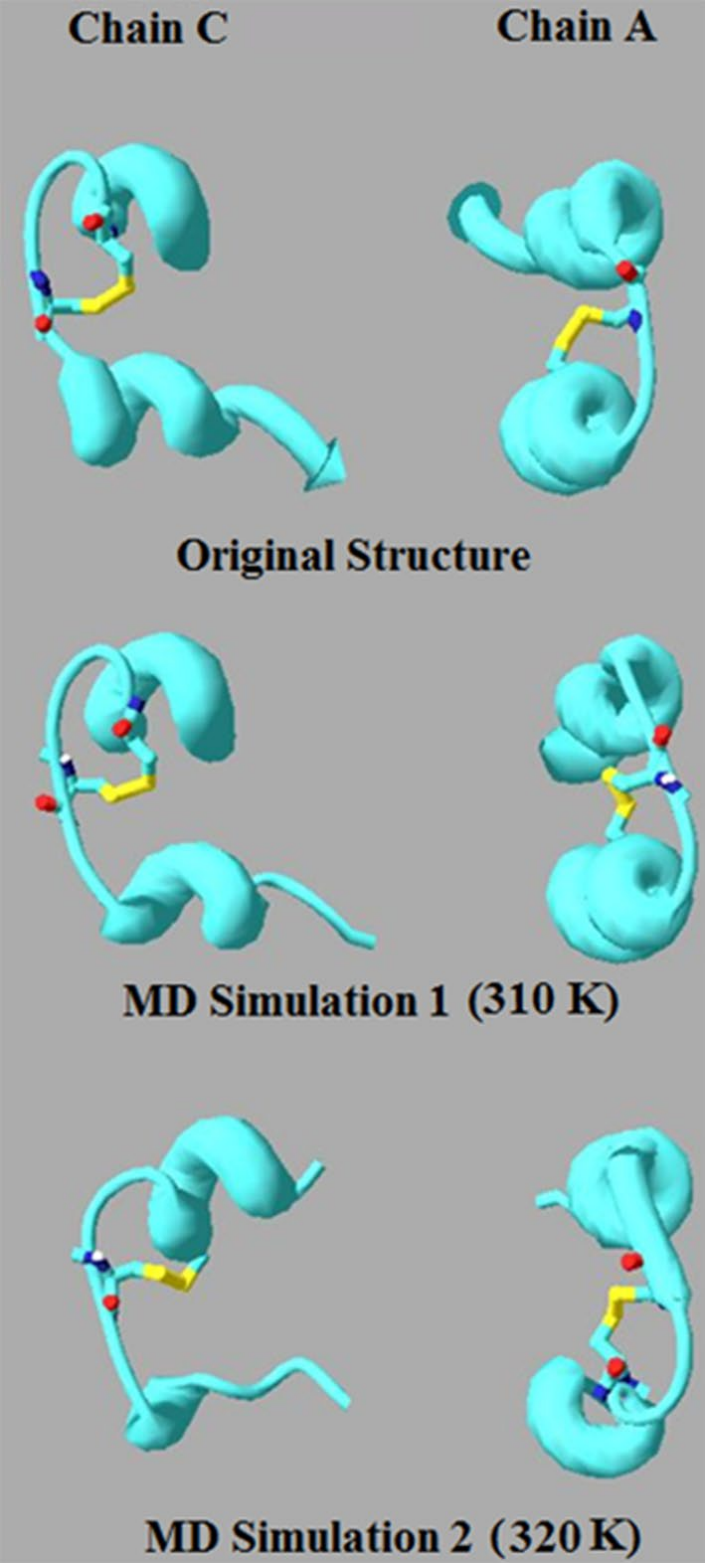
HR insulin(Insugen I) comparison of secondary structures of chains C and A of the major conformation, showing the starting structure (with Cys6–Cys11 S–S bridge shown) and then after MD simulation for 300 ps at 310 and 320 K. Drawn with Swiss-PdbViewer (Deep View) [36]

From a structural point of view, examination of the secondary structures of chains A and C in the original crystal structure of Insugen (I) may provide an explanation for this increased flexibility. The structure of these chains consists of a single loop between two α-helices. The length of the loop shows differences in chains C and A. In chain C the loop is long enough to contain both Cys residues involved in the internal disulphide bridge, whereas in chain A it is shorter and so one of the Cys residues is located on an α-helix. The longer loop of chain C would be more flexible and therefore may possibly allow for more movement of the Cys residues involved in the disulphide bond. Over the course of the MD simulation changes in secondary structure occur, most noticeably in chain C, with significant portions becoming converted to coils, which may further affect flexibility (Fig. 26). In contrast, the results for torsion angle changes and RMSD kinetics for Intergen (II) at 320 K suggest that for this structure, Cys 6–11 of chain A possess greater flexibility than Cys 6–11 of chain A. However, the difference in flexibility would not seem to be as great for Insugen (I).

### Overall conclusions from the molecular dynamics study

From the results of both the torsion angle plots and the RMSD kinetics for the Cys6–Cys11 S–S bonds of Insugen (I), it is clear that both the Cys6–Cys11 internal disulphide bridges in chains A and C of possess flexibility. However the flexibility of Cys6–Cys11 of chain C appears to be much greater, as both the MD simulations for the minor conformation at 310 K and the major conformation show times when the torsion angle of Cys6–Cys11 of chain C shows rapid decreases followed by rapid increases. In contrast, Cys6–Cys11 of chain A only showed one major change, in the major conformation at 310 K, at all other times staying relatively constant. The rapid changes in torsion angles shown by Cys6–Cys11 of chain C of Insugen (I) would appear to explain why it shows disorder in the original crystal structure.

From a structural point of view, examination of the secondary structures of chains A and C of Insugen (I) in the original crystal structure may provide an explanation for this increased flexibility. The structure of these chains consists of a single loop between two α-helices. The length of the loop shows differences in chains C and A. In chain C the loop is long enough to contain both Cys residues involved in the internal disulphide bridge, whereas in chain A it is shorter and so one of the Cys residues is located on an α-helix. The longer loop of chain C would be more flexible and therefore may possibly allow for more movement of the Cys residues involved in the disulphide bond. Over the course of the MD simulation changes in secondary structure occur in Insugen (I), most noticeably in chain C, with significant portions becoming converted to coils, which may further affect flexibility.

## Discussion

### General comments and further selected examples

The ultra-high resolution X-ray structures of two forms of human recombinant insulin, Insugen (I) and Intergen (II), has revealed several quite unexpected and previously unpredicted features. Both Insugen (I) and Intergen (II) structures exhibit structural features that can be described as: (a) highly ordered; (b) clear and resolved double conformations; (c) badly disordered. The assembled molecule comprises polypeptide chains A, B, C and D where A and C are sequence equivalent, as are B and D. It is somewhat surprising that the occurrence of structural features (a), (b) and (c) between say Insugen (I) chains A and C is by no means one to one but rather almost lacking in correspondence. This observation applies to all pairs of like polypeptide chains in both Insugen (I) and Intergen (II) and to all pairs of like polypeptide chains one from Insugen (I) and one from Intergen (II). It would be of interest (1) to find explanations for these differences and (2) to know whether they affect the therapeutic properties of these preparations? As described in “General comments and further selected examples” below why a given residue should be perfectly ordered in one structure and badly disordered in the other? With respect to question (1) the possibilities include (a) method of preparation including folding of the recombinant amino acid-chains and (b) the forces in play when the crystal is cryo cooled prior to X-ray data collection. With respect to question (2) it is well known that differences in the form of a therapeutic insulin preparation with respect to the naturally occurring insulin can induce the production of antibodies in patients. No such indication has been noted with respect to the widespread use of either Insugen (I) or Intergen (II) but is nevertheless a possibility which should be borne in mind. It may be possible to use molecular dynamics simulations further to resolve some of these considerations.

Previous studies: (i) that of Baker et al. [1] at room temperature and a resolution of 1.5 Å on porcine insulin and (ii) that of Smith, Pangborn and Blessing on a commercially available biosynthetic form of T_6_ human insulin (Lilly Research Laboratories) at 120 K and 1.0 Å resolution [24] have revealed significant differences in a number of the individual amino acid residue conformations between the two structures. The level of refinement achieved in these two analyses, as is also the case with the low temperature structure of Intergen (II) HR insulin, as judged by the final R values (0.153, 0.183 and 0.168, respectively) are all inferior to that achieved here with the Insugen (I) HR insulin (0.1112). Interestingly Smith et al. [34] list seven side-chains in the porcine room temperature structure [1] at 1.5 Å resolution and nine side-chains in their 1.0 Å human insulin structure as having two distinct conformations. The residues involved are: porcine **GlnB4**, **ValB12**, GluB21, ArgB22, ThrB27 and LysD29; human **GlnB4**, **ValB12**, GluB17, GluD21, GluC5, LeuC16, ValD12, ValD18 and GluD21. Only two residues are in common in this list: **GlnB4 and ValB12**. This result follows the trend reported here for Insugen (I) and Intergen (II) that correspondence between the two structures with respect to multiple or disordered conformations does not follow any fixed pattern. However, interestingly, reference to Fig. 11 reveals that in both Insugen (I) and Intergen (II) **GlnB4** and **ValB12** presented problems in the interpretation of their electron densities. Of the other residues in the above porcine list some are clear single conformations and others are problematic in either Insugen (I) or Intergen (II). Similar comments apply with respect to the above list for biosynthetic form of T_6_ human insulin. **GlnB4** and **ValB12** are the only residues in all four of these insulin structures that presented problems. According to Fig. 2 **ValB12** is significantly involved in the interaction of insulin with its receptor.

### PheB24 and PheB25 in Insugen (I) and Intergen (II)

**Fig. 27:**
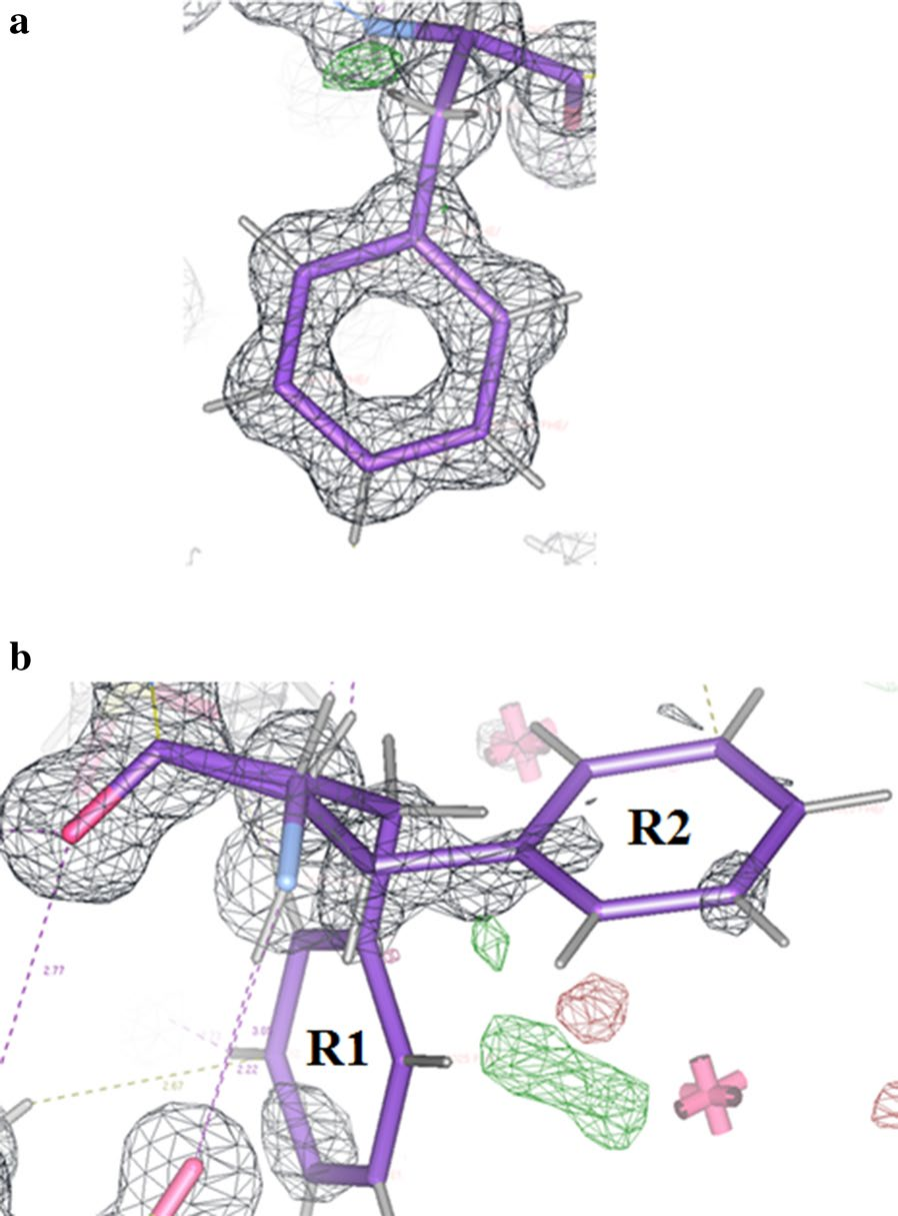
**a** Electron density in the Insugen (I) structure for PheB24, an example of a clear single highly resolved amino acid residue. **b** Electron density in the Insugen (I) structure for PheB25, an example of very poor density modelled as a doubly disordered acid residue. This result is surprising in view of the high order observed in PheB24 (**a**)

**Fig. 28:**
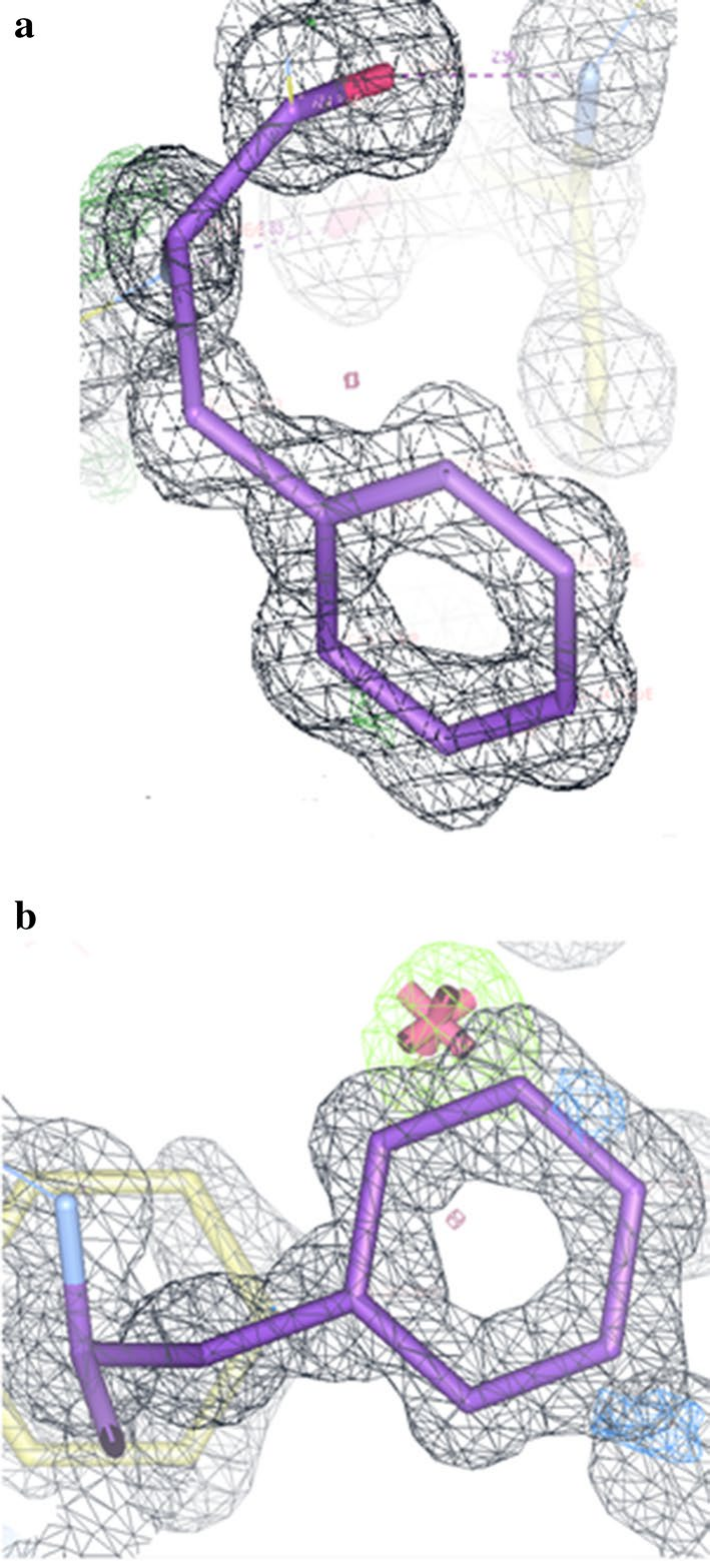
**a** Electron density in the Intergen (II) structure for PheB24, an example of a clear single highly resolved amino acid residue as in Insugen (I), Fig. 27a. **b** Electron density in the Intergen (II) structure for PheB25. Unlike PheB25 in Insugen (I), Fig. 27b which has very weak electron density, this is another example of a clear single highly resolved amino acid residue as in Insugen (I) PheB24, Fig. 27a and Intergen (II) Phe24, (**a**)

A significant example of structural differences in the ultra-high resolution 100 K structures of Insugen (I) and Intergen (II) can be found in the phenylalanine residues PheB24 and B25 (see Fig. 11). As stated previously in “Introduction” these residues, amongst others, are important for insulin receptor binding [6]. As reported by Baker et al. [1] changes in biological activity occur when these residues are modified. Figures 27a, b show the electron density in Insugen (I) for residues PheB24 and B25, respectively while Fig.  28a, b show the electron density in Intergen (II) for the same residues.

The significant observation here is the extremely poor electron density for PheB25 in Insugen (I) which has been modelled as disordered with two distinct conformations, as opposed to PheB24 in Insugen (I) and PheB24 and PheB25 in Intergen (II) which are all excellent examples of strong, clearly resolved single conformation electron density. It is of interest to note that PheB25 in the X-ray structure of porcine insulin [1] has comparatively weak electron density corresponding to a single well defined conformation but with one missing atom on the phenyl ring (see also Footnote 1; Additional file 1: Figure S6a) and PheB24 is in completely well-defined electron density as are PheD24 and PheD25. What is probably most surprising is that while Insugen (I) PheD25 has strong electron density corresponding to a single ordered conformation, as is also the case for Intergen (II) and porcine insulin [2], the conformation for Insugen (I) PheD25 uniquely corresponds to that of the ordered porcine B conformation, not the porcine D conformation as displayed by the other two PheD conformations (see also Additional file 1: Text S6, Figure S6a–h). It is planned to investigate the situation with respect to PheB24 and PheB25 in Insugen (I) and Intergen (II) using molecular dynamics as described in “Molecular dynamics” for the Sγ6–Sγ11 disulphides.

### Intergen (II) structure: chain C(3) S–S bridge between Sγ6–Sγ11

The intra-chain S–S bridge in chain C(3) Cys6–Cys11 has been observed in Insugen (I) to exhibit two ordered conformations. Cys6 occupies a single site while Cys11 occupies two sites with relative occupancies of 0.8 and 0.2, respectively. The geometry and all other refinement characteristics of this bifurcated cysteine bridge are of excellent quality as discussed previously. The corresponding S–S bridge in Insugen (I) chain A(1) is completely ordered which again poses a question about the origin of the distinction between the two molecular dynamics simulations. Molecular dynamics studies have provided rationale in answer to this question. In fact the difference in the disulphides A6–A11 and C6–C11 may be further explained by the difference in solvent exposure. A6 is less than 4 Å from the nearest water solvent molecule and there are several waters modelled in that area which may give greater conformational flexibility to the region. C6 is in a hydrophobic pocket and consequently the disulphide may be more restricted by that environment. This is supported both by the fact that the section of chain B near this part of the molecule is also disordered. With reference to Intergen (II) the corresponding S–S bridge in chain A(1) is also completely singular and ordered. This S–S bridge in chain C(3) as observed by inspection of PDB 3W7Y has been modelled as a single ordered conformation. However, as discussed previously, there is evidence in the electron density (Fig. 16) that this S–S bridge is actually bifurcated as in the corresponding S–S bridge in Insugen (I). S–S bridges with ordered double conformations have been previously reported. For example Cys14–Cys 38 in the ultra-high resolution (0.86 Å) low temperature, synchrotron structure of bovine pancreatic trypsin inhibitor [25] is very similar to Cys6–Cys11 in Insugen (I).

### Solvated propanol

The ultra-high resolution Insugen (I) X-ray structure was found to include an ordered solvated propanol molecule which forms H-bonds with the prominent OG1A of ThrD27 in chain D(4) and two water molecules. The electron density for this solvate is clear and the geometry of the refined propanol is excellent. There is no solvated propanol in Insugen (I) chain B(2) or in either chain B or D in Intergen (II). These differences again offer a challenge to a rational explanation. The origin of the solvated propanol in Insugen (I) may be questioned. However it is known that propanol is a minor component used in the manufacturing process and is most likely to have been introduced into the protein at some stage of the synthesis procedure. To the best of our knowledge no other insulin structure has been shown to include structurally ordered propanol.

### The Zn sites

Insugen (I) and Intergen (II) have been synthesised to include the essential Zn ions present in naturally occurring insulins. The Zn ions are an essential feature in the formation of the crystal structure and are located on a crystallographic three-fold axis.

#### The Zn site in Insugen (I) molecule 1

The electron density in the vicinity of Zn2 in molecule 1 revealed an unexpected feature which was shown to be a solvated acetate molecule. Zn2 is coordinated to both His10B Nε in chain B and an oxygen atom of the acetate. It is most likely that the presence of solvated acetate originated during crystallization. There are no other solvated acetate sites in either Insugen (I) or Intergen (II).

## Additional file

**Additional file 1.** Hexamers in Insugen (I) (PDB 5E7W) and Intergen (PDB 5W7Y), respectively.

## Footnotes

1 In the publication of Baker et al. [1] the pig insulin asymmetric unit is defined as: molecule 1 (chains A1, B1) and molecule 2 (chains A2, B2). For example residue B25.2 Phe refers to phenylalanine 25 in chain B of molecule 2. However in the PDB deposition of this structure, 4INS, molecule 1 is designated by chains A and B, and molecule 2 as chains C and D. All.pdb files referred to in the present publication follow this later format so B25.2 Phe of Baker et al. becomes PheD25. It should also be noted that to the best of our knowledge in this revised format in the deposited pig insulin 4INS.pdb, unexpectedly Baker et al’s molecule 1 corresponds to chains C + D, and molecule 2 to chains A + B. This means that in Baker et al’s Figure 12.2, for example, the left protruding Phe residue which is supposed to be B25.2 Phe (PheD25) is actually B25.1Phe (PheB25). See Supporting Information Figures S6a and S6b produced from the 4INS file for further details. A second example of this interchange of chain designations occurs in Figure 4.12 of Baker et al. [1] which describes ValB12.1 (ValB12) as having a single conformation and ValB12.2 (ValD12) as having two conformations. Inspection of 4INS.pdb however confirms the opposite case with ValB12 having double and ValD12 as having a single conformation. From this it seems to be safe to assume that this interchange of chain designations is consistent throughout Baker et al. Comparisons made in the present publication assume this to be so.
