# Supplementary material for: Ultra-high resolution X-ray structures of two forms of human recombinant insulin at 100 K
Source: Chem Cent J. 2017 Aug 1;11:73. doi: 10.1186/s13065-017-0296-y (PMC5539060; doi:10.1186/s13065-017-0296-y)
Supplement: Supplementary file 1 — Additional file 1. Hexamers in Insugen (I) (PDB 5E7W) and Intergen (PDB 5W7Y), respectively. [file 13065_2017_296_MOESM1_ESM.docx]

**Supporting Information**

**Ultra-High Resolution X-ray Structures of Two Forms of Human Recombinant Insulin at 100 K**

**David. R. Lisgarten^a^, Rex A. Palmer^b*^, Carina M. C. Lobley^c^, Claire E. Naylor^d^, Babur Z. Chowdhry^e^, Zakieh I. Al-Kurdi^f^, Adnan A. Badwan^g^, Brendan J. Howlin^h^, Nicholas C. J. Gibbons^i^,**

**Jose′ W. Saldanha^j^, John N. Lisgarten^k^ and Ajit K. Basak^l^**

(a)Biomolecular Research Group, School of Human and Life Sciences, Canterbury Christ Church University, North Holmes Road, Canterbury, Kent CT1 1QU, UK email [david.lisgarten@canterbury.ac.uk](mailto:david.lisgarten@canterbury.ac.uk) +44(0)1227782356

(b) Department of Crystallography, Biochemical Sciences, Birkbeck College, Malet St, London WC1E7HX, UK email [rex.palmer@btinternet.com](mailto:rex.palmer@btinternet.com) +44( 0)2084491049

(c) Diamond Light Source Ltd, Diamond House, Harwell Science and Innovation Campus, Didcot, Oxfordshire, OX11 0DE email [carina.lobley@diamond.ac.uk](mailto:carina.lobley@diamond.ac.uk) : +44(0)1235778722.

(d) Molecular Dimensions Ltd, Unit 6, Goodwin Business Park, Willie Snaith Road, Newmarket, Suffolk, CB8 7SQ email Claire@moleculardimensions.com

(e) School of Science, University of Greenwich (Medway Campus), Chatham Maritime,

Kent ME4 4TB, UK email [b.z.chowdhry@greenwich.ac.uk](mailto:b.z.chowdhry@greenwich.ac.uk) +44(0)2083318208

(f)  The Jordanian Pharmaceutical Manufacturing Company (PLC), Suwagh Subsidiary for Drug Delivery Systems, P.O. Box 94, Naor 11710, Jordan. email [zkurdi@jpm.com.jo](mailto:zkurdi@jpm.com.jo)

(g) The Jordanian Pharmaceutical Manufacturing Company (PLC), Suwagh Subsidiary for Drug Delivery Systems, P.O. Box 94, Naor 11710, Jordan. email dr.badwan@jpm.com.jo (h) Chemical Sciences Division, Faculty of Health and Medical Sciences, University of Surrey, Guildford, Surrey GU2 7HX, UK email b.howlin@surrey.ac.uk+44(0)1483300800.

(i) Department of Natural Sciences, School of Science and Technology, University of Middlesex, Hendon Campus, The Burroughs, London, NW4 4BT, UK email n.gibbons@mdx.ac.uk

(j) MRC National Institute for Medical Research, The Ridgeway, Mill Hill, London NW71AA email [jsaldan.nimr@gmail.com](mailto:jsaldan.nimr@gmail.com)

(k) School of Science, University of Greenwich (Medway Campus), Chatham Maritime,

Kent ME4 4TB, UK email jlisgarten@hotmail.com

(l) Department of Crystallography, Biochemical Sciences, Birkbeck College, Malet St, London WC1E7HX, UK email [a.basak@mail.cryst.bbk.ac.uk](mailto:a.basak@mail.cryst.bbk.ac.uk) +44(0)02076316823

*Corresponding Author

**
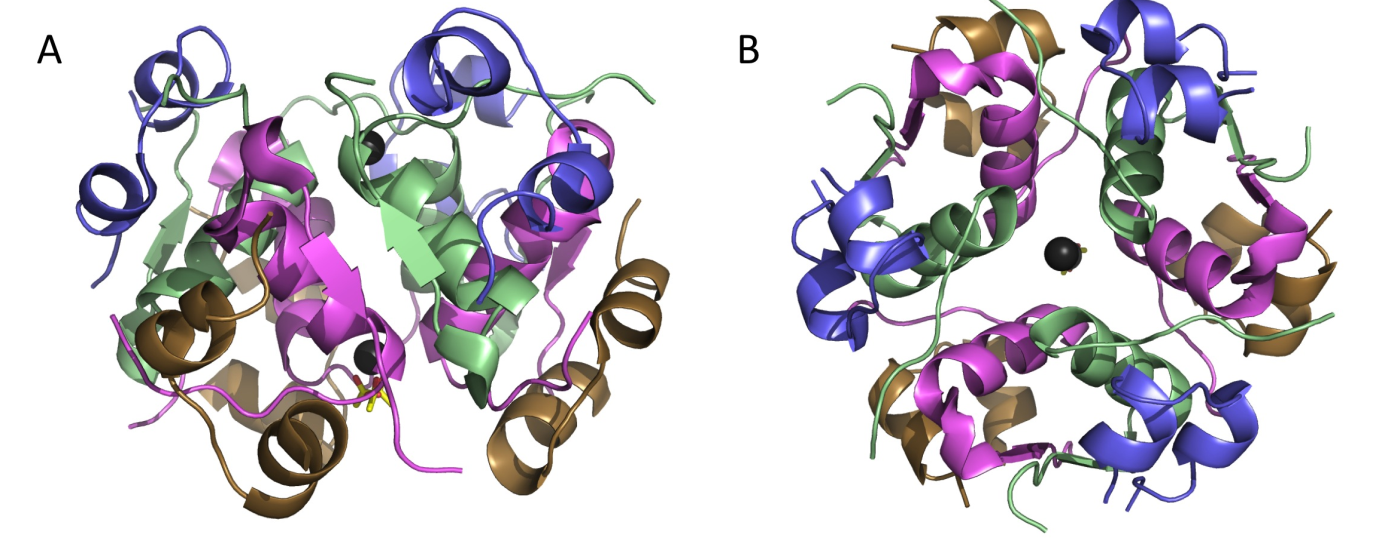
**

**
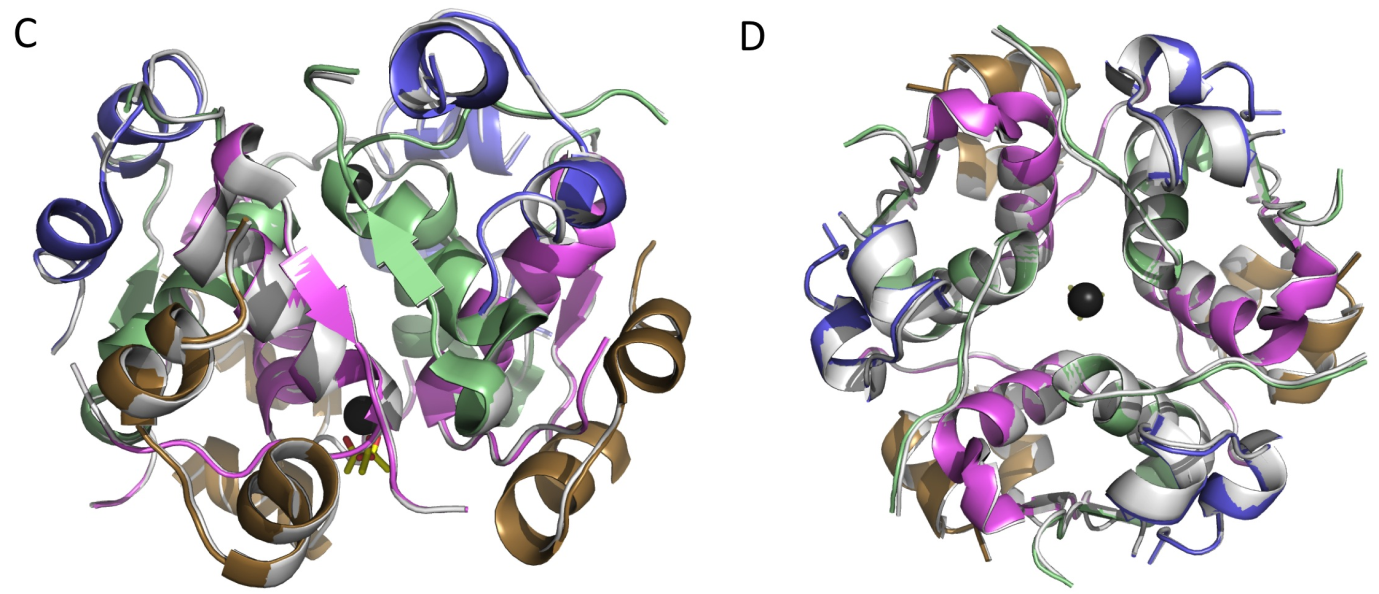
**

**Figure S1. A and B are two perpendicular views of the Insugen(I) hexamer. Colours: chain A sand; chain B magenta; chain C slate; chain D pale green; Zinc ions black; acetate yellow sticks. Similarly C and D are Intergen(II) 3W7Y in white shown with Insugen(I) coloured as for A and B. Drawn with the PyMOL Molecular Graphics System, Version 1.7.4 Schrödinger, LLC.**


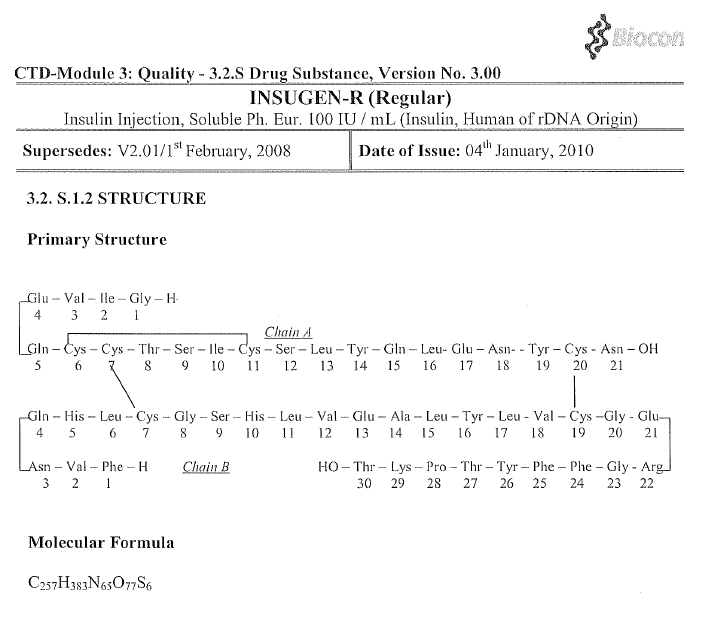


**Figure S2a. Product description for Insugen(I).**


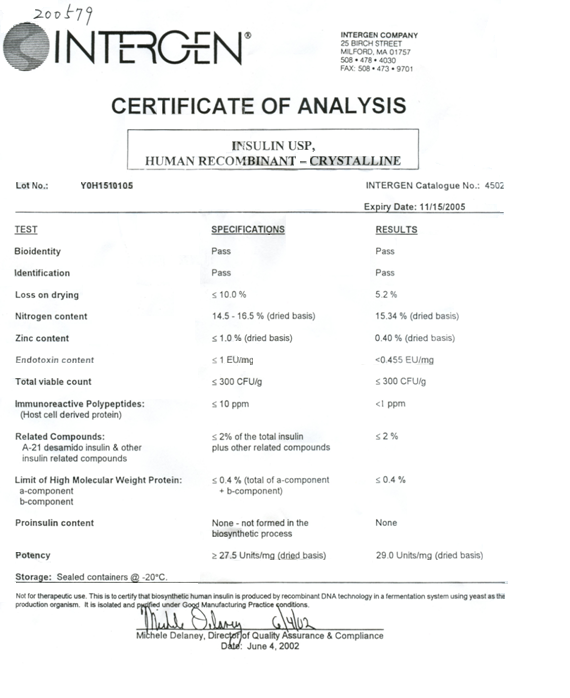


**Figure S2b. Product description for Intergen(II).**

**Table S1. Additional Refinement Statistics for Insugen(I).**

Structure Solution Molecular Replacement

ReflnsShelX List 40.91-0.92 (0.94-0.92)

Resolution (Low) 40.91

Number of Reflections (Observed) 294902 (13731 outer shell)

(Unique) 58647 (2895 outer shell)

Number of Reflections (R-Free) 55679 (5.0592%)

Reflections in R-Free 2968

Percent Reflections (Observed) 100 (100 outer shell)

Multiplicity 5.0 (4.7 outer shell)

R-Factor (Observed):

R-Work 0.108

R-Free 0.146

R-Free Selection Details 5% of all data

Temperature Factor Model:

Mean Isotropic B Value 17.947

Refinement Method SHELXL

Shell Resolution (High) 0.952

Shell Resolution (Low) 13.637

Number of Reflections (Observed):

Number of Reflections (R-Free) 2692

Number of Reflections (R-Work) 50178

R-Factor (R-Work) 0.1201

R-Factor (R-Free) 0.1420

Percent Reflections (Observed) 5%

Number of Non-Hydrogen Atoms Used in Refinement

Protein Atoms 1729

Heterogen Atoms 8

Solvent Atoms 220

**Text S1: Zinc Co-ordinating Residues in Insugen(I)**

The bound zinc sites in both the B and D chains are shown in Supplementary Figure S3. In both cases the site is on the three-fold axis and so exhibits trigonal symmetry: one side of the zinc is co-ordinated in both cases by three symmetry equivalent copies of His10 from the B and D chains respectively. The electron density for the zinc co-ordinating residues on the other side of the zinc ion is distinctively different for the two chains, however. The B chain associated site is shown in Supplementary Figure S3 A and B: It shows three strong, clear, spherical peaks well accounted for by three symmetry-related, fully-occupied water molecules. This results in an octahedral co-ordination unusual for zinc. The electron density for non-protein zinc-coordinating ligands in chain D, shown in Supplementary Figure S3 C and D, is far more complex, however, revealing a series of smaller peaks, too close to one another to represent a water network. Inspection of the crystallisation conditions indicates the presence of acetate. The three-fold symmetry present means that the acetate must occupy each of three symmetry equivalent sites, but simultaneous occupation of these sites results in physically impossible overlapping of two molecules: therefore in any one molecule only a single acetate will be present resulting in a bulk occupancy of one-third for each symmetry-related acetate molecule. This model fits the observed electron density well, and since it results in a single oxygen atom co-ordinating the zinc in each case it also provides a distorted tetrahedral ligand geometry more commonly observed for zinc ions. However, the low occupancy of light atoms (one third of carbon’s six electrons corresponds to a peak height of 2e) results in only weak electron density for the acetate molecules in the refined model.

**
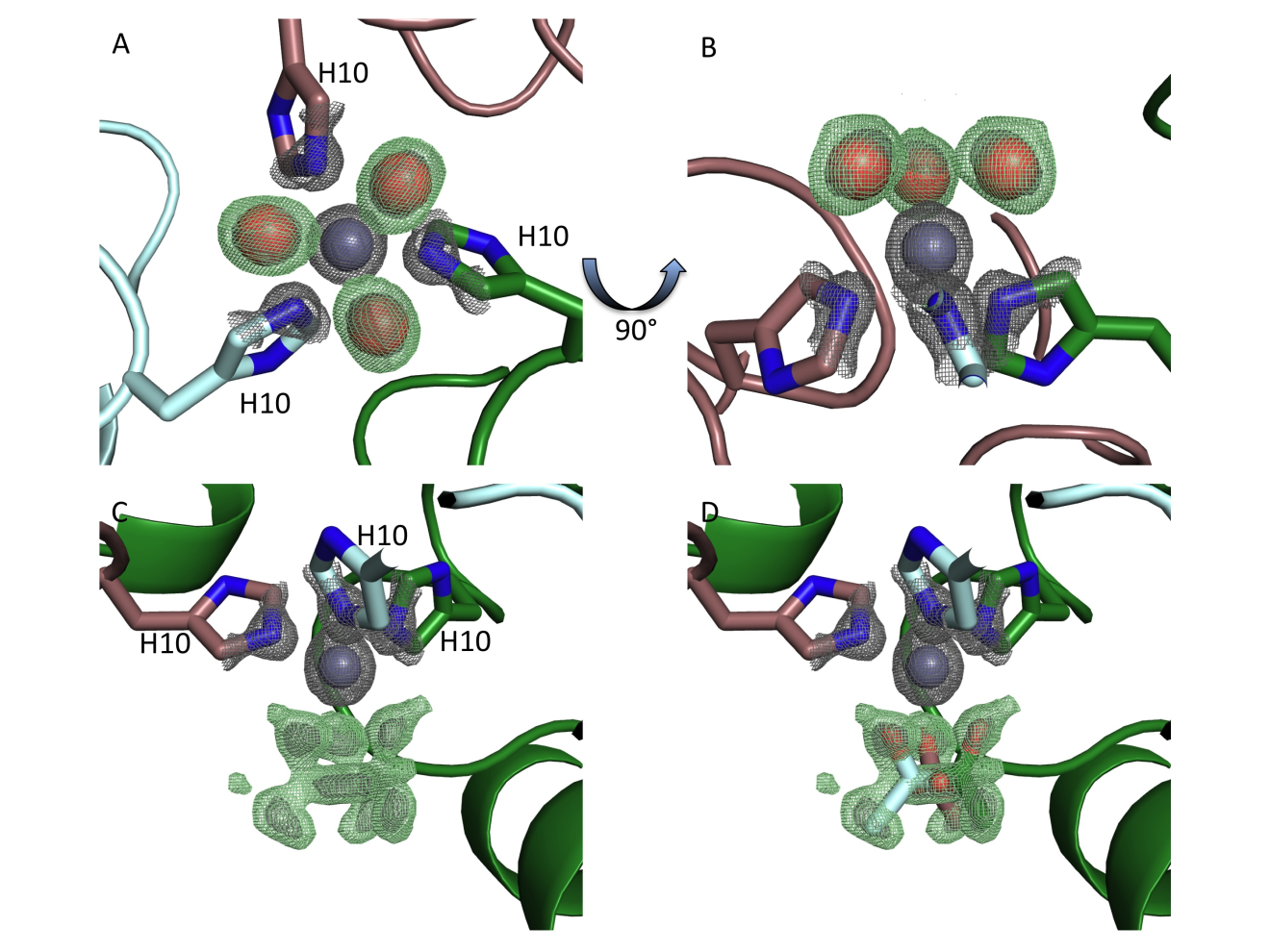
**

**Figure S3.** In all panels, insulin is drawn as cartoon in light blue, green and pink and the exception of His10 from each chain is shown as sticks. Zinc ions drawn as grey spheres. The zinc site is at a symmetry axis and so all three chains crystallographically identical. Electron density calculated with phases omitting the contribution of the non-protein zinc co-ordinating ligands is shown as grey mesh for a 3F_o_-2F_c_ map contoured at 1.5 rmsd and as green mesh for a difference map contoured at 5.0 rmsd. Panels (A) and (B) show B chain associated zinc sites with co-ordinating water as a red spheres, at two orthogonal views for clarity. Panels (C) and (D) show the D chain associated site: (C) without the acetate ligand drawn to allow a clear view of the density and (D) with all three symmetry equivalent and partially occupied acetates drawn as sticks.

**Text S2: Evidence in Support of the Assignment of Propanol in Insugen(I)**

At the end of the SHELXPRO [22] refinement a significant region of unaccounted for density remained in the cleft between the N-terminus of the C chain and the C-terminus of the D chain, Supplementary Figure S4(a). This density was both too extensive and too strong to be easily accounted for by multiple waters. A number of different molecules were modelled into the density in an attempt to adequately explain it. In Supplementary Figure S4(b) residue Gly1 of the A chain has been replaced with a lysine side chain. The proximity of the density to Gly1 and its shape make this the most convincing fit to the density, and this is reflected both in a slightly lower R-factor and the best RSR scores for all the options. However, there is no other evidence to suggest that a Gly1-Lys1 mutation has occurred and so it was discounted on this basis. Inspection of the contents of the cryo- and crystallisation conditions revealed the presence of glycerol, which is often identified binding to proteins non-specifically in high resolution protein crystal structures. However, modelling of glycerol into the density, Supplementary Figure S4(c), indicated that this molecule was too large for the small cavity and resulted in a major clash with the Gly1 main chain. In addition, there was no density of any sort for the central glycerol hydroxyl, despite the fact that both ends of the molecule are well localized so that this hydroxyl should have little or no possibility for disorder. Finally a propanol was modelled into the density, Supplementary Figure S4(d). This molecule fitted well into the density (almost as well as the Gly1-Lys1 mutation), and explains the observed density well, has good molecular geometry and makes a good hydrogen bond. However, it has not been possible to determine when or if the protein has been exposed to propanol (but see [28]). Further details of the refined propanol model are given in the main text (Section 4.6.2), the geometry of the modelled propanol and its interactions with the protein.


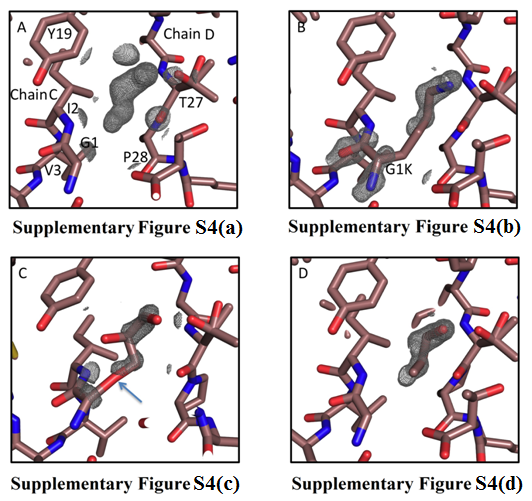


**Figure S4:** HRInsulin Insugen(I) shown in the same orientation drawn in dusky pink sticks in each panel with 3F_o_-2F_c_ maps shown at 1.5 rmsd as grey mesh. A chain on the left and B chain on the right hand side of each panel. (a) Density with model omitted for the additional density, surrounding residues labelled for information. (b) Refinement with Gly1 replaced with Lys. (c) Refinement with glycerol modelled into the density. Clash with Gly1 main chain indicated with arrow. (d) Refinement with propan-(1)-ol placed in the density.

**Table S3.**

**Survey of individual 0.92Å resolution electron density of the amino acid residues in polypeptide chains A, B, C and D in Insugen(I) and Intergen(II).**

**Abbreviations used:**

oc = ordered clear density

pd = problems with density fit

cd = clear disorder in two parts and modelled in refined structure

md = missing density according to expected model

**Table S3(a).**

**Insugen(I) chain A Intergen(II) 3W7Y chain A**

G1 oc oc

I2 oc oc

V3 oc oc

E4 oc oc

Q5 oc oc

C6 – C11 oc oc

C7 oc oc

T8 oc oc

S9 oc oc

I10 oc oc

C11 See C6

S12 oc oc

L13 oc oc

Y14 oc oc

Q15 oc oc

L16 oc oc

E17 oc oc

N18 oc oc

Y19 oc oc

C20 oc oc

**N21 cd + md at terminus oc**

**Note: Major differences are indicated in bold**

**Table S3(b).**

**Abbreviations used:**

oc = ordered clear density

pd = problems with density fit

cd = clear disorder in two parts and modelled in refined structure

cdnm = apparently disordered in two parts but modelled as a single chain

md = missing density according to expected model

**Insugen(I) chain B Intergen(II) 3W7Y chain B**

F1 oc oc but md at end of residue

V2 oc oc

N3 oc oc

Q4 cd but some md oc but md at end of residue

H5 oc oc

L6 oc oc

C7 oc oc

G8 oc oc

S9 oc oc

H10 oc oc

**L11 cd cdnm**

**V12 cd cdnm**

**E13 cd oc**

A14 oc oc

L15 oc oc

Y16 oc oc

**L17 cd + some md oc**

V18 oc oc

C19 oc oc

G20 oc oc

**E21 cd oc**

**R22 cd oc disordered in Baker *et al* [1]**

G23 oc oc

F24 oc oc see Figures 20(a,c)

**F25 pd + md in ring atoms oc** see Figures 20(b,c)

Y26 oc oc

**T27 cd cd but some md**

P28 oc oc

**K29 oc + md oc**

T30 oc but some md oc but some md

**Note: Major differences are indicated in bold**

**Table S3(c).**

**Abbreviations used:**

oc = ordered clear density

pd = problems with density fit

cd = clear disorder in two parts and modelled in refined structure

md = missing density according to expected model

**Insugen (1) chain C Intergen(II) 3W7Y chain C**

G1* oc oc

I2 oc oc

V3 oc oc

E4* oc oc

Q5 mainly oc but weak at end oc but md at end

**C6–C11 cd oc modelled but possibly cd**

C7 oc oc

T8 oc oc

**S9 oc oc modelled but possibly cd**

I10 oc **oc modelled but possibly cd**

C11 See C6

S12 oc oc

L13 oc oc

**Y14 oc +pd at end on OH pd fitted as Ala not Tyr**

Q15 pd + md disordered pd disordered see Figures Xc,d

**L16 cd oc**

E17 oc oc

N18 oc **oc modelled but possibly cd**

Y19 oc oc

C20 oc oc

N21* oc oc

**Note: Major differences are indicated in bold**

**Table S3(d).**

**Abbreviations used:**

oc = ordered clear density

pd = problems with density fit

cd = clear disorder in two parts and modelled in refined structure

md = missing density according to expected model

**Insugen(I) chain D Intergen(II) 3W7Y chain D**

**F1 md oc** **but weak**

**V2 pd oc**

N3 oc oc

**Q4 very poor pd oc but md at end**

H5 oc oc

L6 oc oc

C7 oc oc

G8 oc oc

S9 oc oc

H10 oc oc

L11 oc oc

**V12 cd oc modelled but possibly cd**

E13 oc oc

A14 oc oc

L15 oc oc

Y16 oc oc

L17 oc oc

**V18 cd oc**

C19 oc oc

G20 oc oc

E21 pd missing atoms pd + md

R22 oc oc

G23 oc oc

F24 oc oc

F25 oc oc

Y26 oc oc

**T27 cd some missing atoms oc**

P28 oc oc

K29 pd missing atoms pd missing atoms

T30 oc oc

**Notes:**

**1. Major differences are indicated in bold. 2. E21 and R22 are also disordered in porcine insulin (Baker *et al*)**


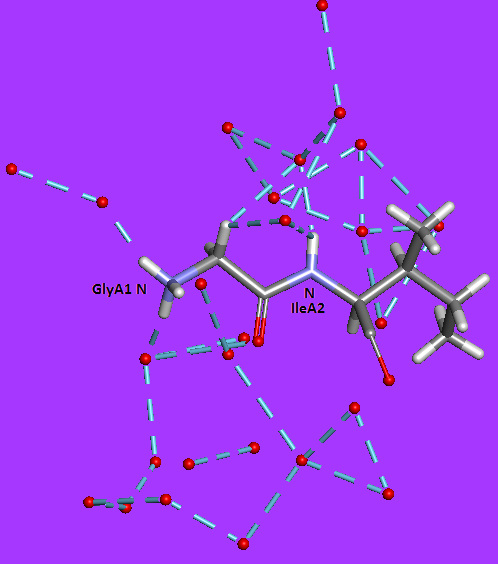


**Figure S3a. Insugen(I) human recombinant insulin GlyA1 and IleA2 showing the stabilizing water H-bond network. 18 waters are involved. Hydrogen bonds are shown. Drawn with Accelrys [26a].**

**
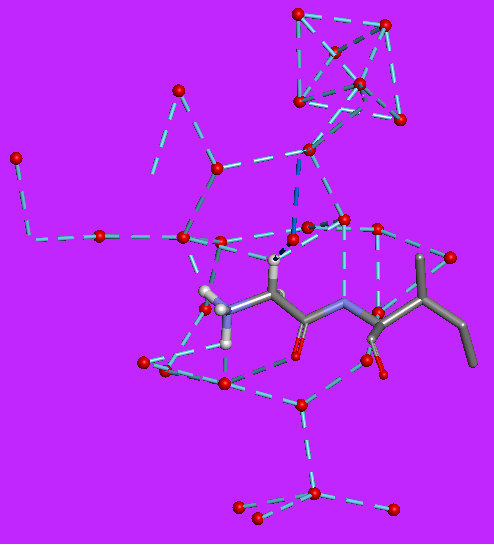
**

**Figure S3b. As for Figure S3a but for Intergen(II) human recombinant insulin (3W4Y) - GlyA1 and IleA2 showing the stabilizing water H-bond network similar to that for Insugen (Figure S3a). Hydrogen bonds are shown. Drawn with Accelrys [26a].**


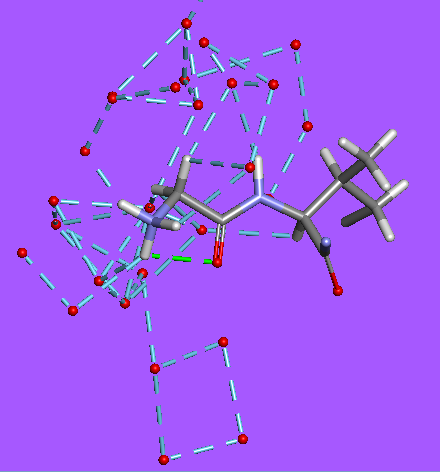


**Figure S3c. As for Figure S3a but for porcine insulin (4INS)-GlyA1 and IleA2 showing the stabilizing water H-bond network similar to that for Insugen (Figure S3a). Hydrogen bonds are shown. Drawn with Accelrys [26a].**

**
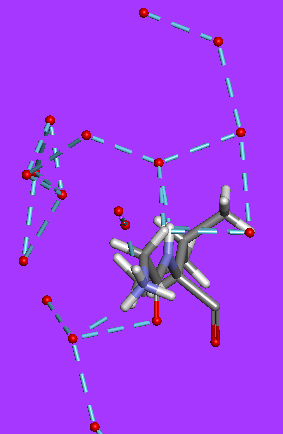
**

**Figure S3d. Insugen(I) human recombinant insulin GlyA1 and IleA2 viewed from GlyA1 to IleA2 showing the stabilizing water H-bond network. Hydrogen bonds are shown. Drawn with Accelrys [26a].**

**
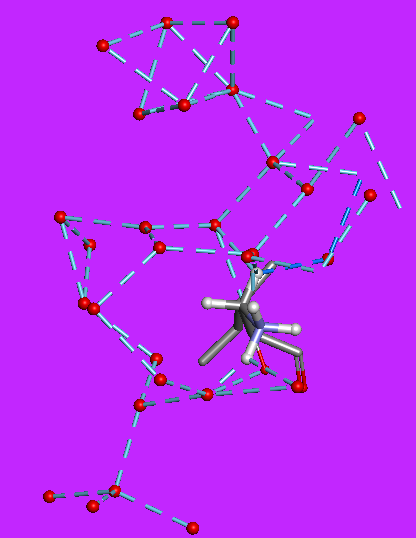
**

**Figure S3e. Intergen(II) human recombinant insulin - GlyA1 and IleA2 viewed from GlyA1 to IleA2 showing the stabilizing water H-bond network. Hydrogen bonds are shown. Drawn with Accelrys [26a].**

**
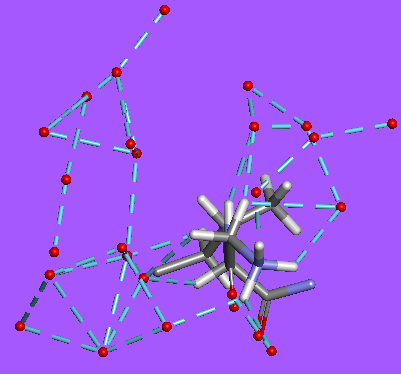
**

**Figure S3f As for Figure S3d but for porcine insulin (4INS) again viewed from GlyA1 to IleA2 showing the stabilizing water H-bond network. Hydrogen bonds are shown. Drawn with Accelrys [26a].**

**
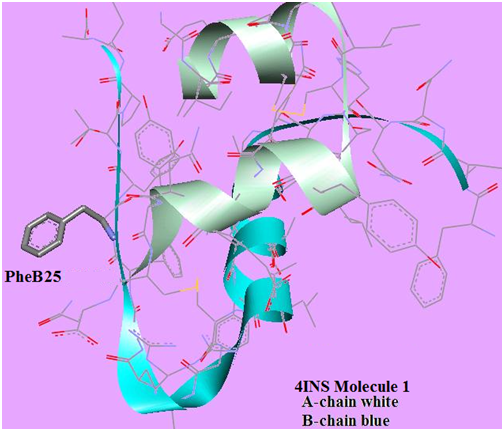
**

**Figure S4(a). See Footnote 1. Drawn with Accelrys [26a]**

**
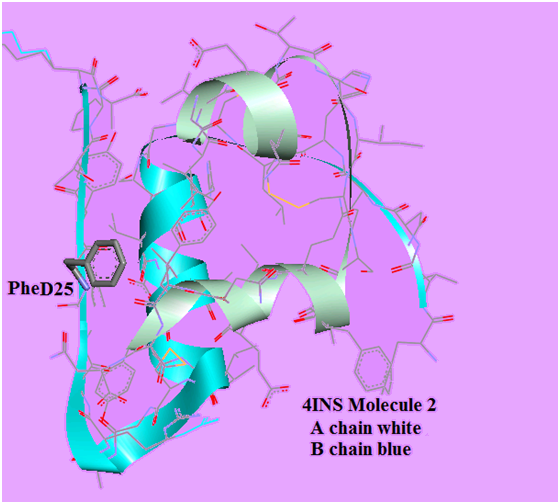
**

**Figure S4 (b). See Footnote 1. Drawn with Accelrys [26a]**


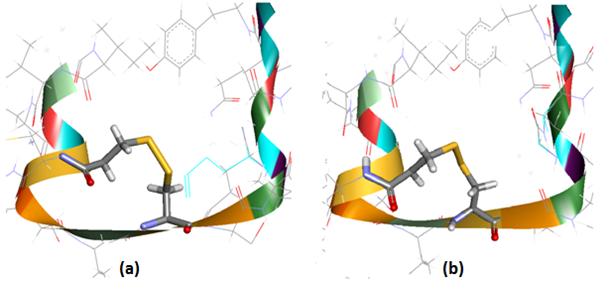


**Figure S5. Insugen(I) structure: chain C(3) S-S bridge between Sγ6 - Sγ11 showing the geometry of (a) the major conformation part A and (b) the minor conformation part B of the disordered S-S bridge in chain C(3). Drawn with Accelrys Discovery Studio 3 [26a].**

**
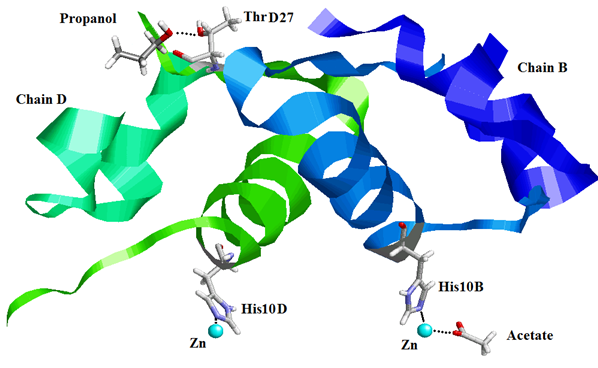
**

**Figure S8. Insugen(I) human recombinant insulin showing the Zn sites in chains B and D. In chain B Zn is coordinated to both a His residue and an acetate moiety and in chain D to a His residue only. The propanol molecule associated with ThrD27 is also shown. Drawn with Accelrys (Discovery) software [26a].**

**
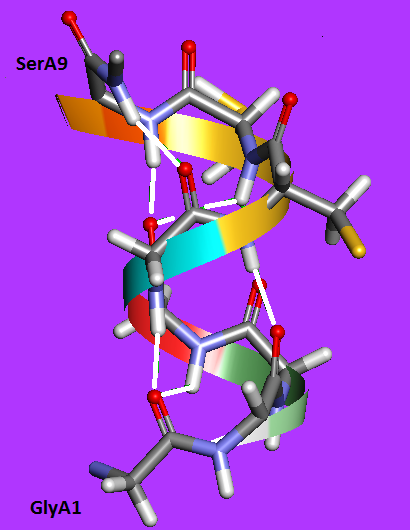
**

**Figure S9(a). Porcine insulin helix A1: GlyA1 – SerA9 showing main chain atoms only. Hydrogen atoms were inserted geometrically. H-bonds are shown as white lines.** **Drawn with Accelrys Discovery Studio 3 [26a].**  **Compare with Figure 4(b) which shows the same region for Insugen(I).**

**
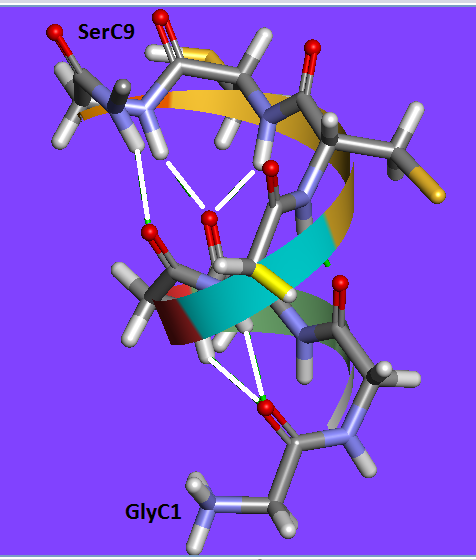
**

**Figure S9(b). Porcine insulin helix C1: GlyA1 – SerA9 showing main chain atoms only. Hydrogen atoms were inserted geometrically. H-bonds are shown as white lines.** **Drawn with Accelrys Discovery Studio 3 [26a]. Compare with Figure 4(e) which shows the same region for Insugen(I).**
